# Ecological succession and the competition-colonization trade-off in microbial communities

**DOI:** 10.1186/s12915-022-01462-5

**Published:** 2022-11-30

**Authors:** Miles T. Wetherington, Krisztina Nagy, László Dér, Ágnes Ábrahám, Janneke Noorlag, Peter Galajda, Juan E. Keymer

**Affiliations:** 1 Department of Ecology, School of Biological Sciences, P. Catholic University of Chile, Santiago, Chile; 2 Biological Research Centre, Institute of Biophysics, Szeged, Hungary; 3 School of Applied and Engineering Physics, Cornell University, Ithaca, USA; 4 Doctoral School of Multidisciplinary Medical Sciences, University of Szeged, Szeged, Hungary; 5 Institute of Physics, School of Physics, P. Catholic University of Chile, Santiago, Chile; 6 Department of Natural Sciences and Technology, University of Aysén, Coyhaique, Chile

**Keywords:** Ecological succession, Competition-colonization trade-off, Microbial landscape ecology, Metacommunities, Microfluidics

## Abstract

**Background:**

During range expansion in spatially distributed habitats, organisms differ from one another in terms of their patterns of localization versus propagation. To exploit locations or explore the landscape? This is the competition-colonization trade-off, a dichotomy at the core of ecological succession. In bacterial communities, this trade-off is a fundamental mechanism towards understanding spatio-temporal fluxes in microbiome composition.

**Results:**

Using microfluidics devices as structured bacterial habitats, we show that, in a synthetic two-species community of motile strains, *Escherichia coli* is a fugitive species, whereas *Pseudomonas aeruginosa* is a slower colonizer but superior competitor. We provide evidence highlighting the role of succession and the relevance of this trade-off in the community assembly of bacteria in spatially distributed patchy landscapes. Furthermore, aggregation-dependent priority effects enhance coexistence which is not possible in well-mixed environments.

**Conclusions:**

Our findings underscore the interplay between micron-scale landscape structure and dispersal in shaping biodiversity patterns in microbial ecosystems. Understanding this interplay is key to unleash the technological revolution of microbiome applications.

**Supplementary Information:**

The online version contains supplementary material available at 10.1186/s12915-022-01462-5.

## Background

The goal of microbial ecology is to understand the mechanisms which maintain biodiversity [1] and determine species distributions [2]. The need to understand increases as we learn more about the spatial subtleties of bacterial worlds and their roles, working as microbiomes present in both our bodies and the ecosystems we rely on [3–5]. Research on spatial biology has been directed towards understanding how the structure of habitats determines ecological processes. For example, How does structure affect colonization-extinction dynamics? How do these processes shape communities coupled via dispersal? Does patchiness permit coexistence? A myriad of potential frameworks, both niche [6] and neutral [7], can be used to address these questions. One of the lessons has been that there is not one silver bullet for maintaining biodiversity, but rather many potential causes for coexistence [8]. These depend on specific details of the system, for example, species pools [9], ephemerality [10] and structure of the habitat [11]. In fact, for bacteria, the spatial structure is of paramount importance as their habitats are porous and patchy.

The structure of bacterial habitats as well as the ephemerality of the species inhabiting them play an important role in determining community composition, yet they are rarely considered experimentally [12]. Ecological theory, such as metacommunity theory [8] and ecological succession [13], is necessary for addressing these questions in microbiology. It is undisputed that microbes are constantly regulating and responding to their environment, through biofilm formation [14], collective migration [15], consumption of nutrients, and excretion of metabolites in ways that have an immediate effect on habitat quality (ecological inheritance) and thus ecological selection [16]. Niche construction of this kind plays a role in ecological succession in microbial landscapes where the stochastic nature of colonization can dictate its dynamics (priority effects) [17]. This is particularly important in environments where succession occurs rapidly and dispersal capabilities play an important role in determining the order of arrival [18]. A variety of microbial ecosystems fall into this category, including human microbiomes such as gut and skin [19, 20], the phyllosphere (leaf surfaces) [13], and the rhizosphere (soil-root interface) [21]. Predicting how microbe-environment coupling shapes composition is still an open question. To progress, it is essential to link the structure of the habitat with the biology of populations and the ecology of communities [22]; microbiomes are no exception [23].

**Fig. 1:**
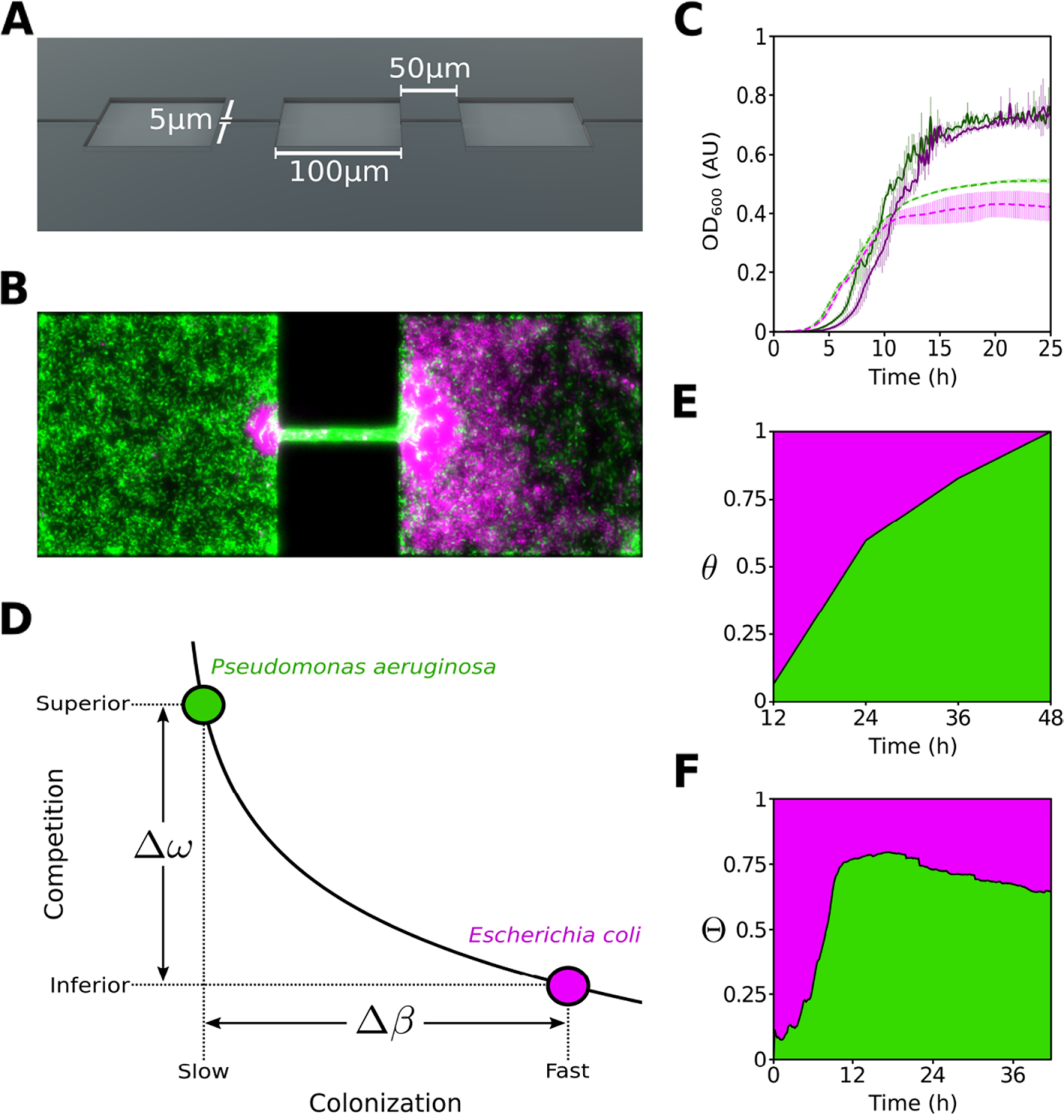
Competition-colonization trade-off in micro-fabricated habitats. **A** Schematic showing a section of a patchy landscape. **B** Fluorescence microscopy image of the patch-corridor motif depicting *E. coli* (magenta) competing against *P. aeruginosa* (green). **C** Monoculture growth of *P. aeruginosa* strains (solid line; PUPa3-G in green and PUPa3-R in magenta) and *E. coli* strains (dashed line; JEK1036 in green and JEK1037 in magenta) in well-mixed 96 well-plates. **D** Two ecological strategies. Curve represents Equation 1 ($\alpha =4$) describing the trade-off. **E** Community dynamics in well-mixed environments represented as *P. aeruginosa* fractional occupancy, $\theta \equiv \langle N_P/(N_E+N_P)\rangle$; $N_P$ and $N_E$ represent cell counts of P. aeruginosa and *E. coli* respectively (see 5). *P. aeruginosa* to *E. coli* initial inoculation ratio 1:1. **F** Metacommunity dynamics in patchy landscapes represented as *P. aeruginosa* fractional occupancy, $\Theta \equiv \langle \bar{P}/(\bar{E}+\bar{P})\rangle$, calculated as an ensemble average by integrating all 72 landscapes; $\bar{P}$ and $\bar{E}$ represent the spatial average occupancy of *P. aeruginosa* and *E. coli*, respectively

In this work, we studied ecological succession in a bacterial meta-community of *Escherichia coli* and *Pseudomonas aeruginosa* in spatially distributed habitats structured at the micron-scale (see Fig. 1). Building upon previous work [24, 25], we focus on: (*i*) in what order do the species colonize the landscape? (*ii*) how does this impact composition? and (*iii*) are metacommunity dynamics reproducible?

We found that the order of colonization is reproducible, where *E. coli* and *P. aeruginosa* demonstrate a competition-colonization trade-off as *E. coli* colonizes first (fast colonizer) followed by succession by the superior competitor, *P. aeruginosa* (slow colonizer). In patchy habitats (Fig. 1A), niche construction by *E. coli* within ecological corridors effectively limits dispersal (Fig. 1B) and leads to fragmentation events preventing the superior competitor from dominating. These processes determine three temporal scales that can be appreciated when measuring the ensemble average dynamics of *P. aeruginosa*’s fractional occupancy; a proxy for community structure (Fig. 1F). In habitats with no patchiness, coexistence is based on a rather neutral ecology while in well-mixed environments it is not possible (Fig. 1E and Additional file 1: Fig. S1). Our observations are explained by a spatially-explicit stochastic model which decouples interference from colonization (scrambling).

## Results

### Landscape ecology *on-chip*

Patchiness introduces a critical length scale in the landscape [22]. This determines the existence of fugitive strategies [26] which specialize in scramble competition for vacancy as opposed to excelling at interference competition within patches. To be or not to be a fugitive strategy? This is the core of the competition-colonization (CC) trade-off. This dichotomy is sketched in Fig. 1D, a relationship [27] linking competition ($\omega$) and colonization ($\beta$) abilities with $\alpha$ indicating its strength (see Additional file 1: Table S1 for symbols),

1 $$\begin{aligned} \omega = e^{-\alpha \beta }. \end{aligned}$$

If patchiness exists, *E. coli* and *P. aeruginosa* can be positioned along this space of differentiation. The strategy of *E. coli* corresponds to a *fast* colonizer (higher $\beta$) and *P. aeruginosa* to a *superior* competitor (higher $\omega$). On the contrary, if the habitat is not patchy, the dichotomy vanishes and so do these phenotypes. To imitate the patchy nature of microbial habitats [28], we used microfabrication [29] to generate landscapes (see Additional file 1: Table S2 for glossary) where arrays of habitat patches are connected by corridors [24]. We fabricated 85 repetitions of a patch-corridor motif (Fig. 1A and Additional file 1: Fig. S2A) giving rise to a one dimensional crystal-like array of microhabitat patches (MHPs). For comparison, we made “flat landscape” environments which consist of a single, long MHP making up a strip-like habitat.

In these devices, we performed invasion-competition experiments using time-lapse microscopy of fluorescently labeled (RFP–GFP and GFP–RFP) co-cultures of wild-type strain pairs (*E. coli*–*P. aeruginosa*, shown here as magenta–green) to study ecological succession as the community develops over 48 hours (see Additional file 1: Table S3 for strains). We initiated the system with each species invading from opposite sides and recorded images in 10-min intervals (alternative initial conditions were also considered, see Additional file 1: Fig. S3 and Additional file 2: Supplementary Note 1). Local, within MHP (spatial index *k*), occupancy was calculated for both *P. aeruginosa*, $P_k(t)$, and *E. coli*, $E_k(t)$, for all images (time index *t*). Figure 1F shows *P. aeruginosa* fractional occupancy dynamics, ${\Theta }(t)$, calculated as an ensemble average by combining the data of 72 patchy landscapes (24 experiments containing 3 landscapes each). Community dynamics can be broken down in three stages: (i) a quick colonization by *E. coli* followed by (ii) an increasing domination by *P. aeruginosa* reaching a relative occupancy of ${\Theta } \sim 0.8$ 12 h after inoculation and (iii) a steady relaxation to a more moderate, albeit still *P. aeruginosa* dominated, coexistence; $\Theta \rightarrow 0.65$. This is in striking contrast with results in well-mixed flasks where a steady increase in *P. aeruginosa* fractional abundance $\theta (t)$ was observed (Fig. 1E).

### Population waves and monoculture metapopulations

Bacterial waves have been described since the seminal work of Adler [30] and their existence is a robust and integral part of bacterial dispersal [15, 24, 25, 30–32]. They are the product of interactions between attractant fields and the chemotactic behavior of individuals [30, 33]. These interactions lead to directional persistence in the swimming patterns of cells [15]. Waves drive quorum sensing [32] guided by the topology of the habitat [31]; thus, we expect them to set the conditions for coexistence (Fig. 1F). Previous work on monocultures colonizing similar landscapes has shown that after initial colonization by waves [25], bacteria develop into metapopulations [24]. Here, we link these waves to a mechanism promoting biodiversity by allowing persistence of a fugitive strategy [26].

In Fig. 1C, we show that in well-mixed monocultures *E. coli* is faster than *P. aeruginosa* at exiting lag-phase. This difference may be interrelated to wave formation and propagation. As the bacteria expand their range, waves experience diffraction [25] and produce a complex phenotype of patch colonization and local extinction (see Additional file 1: Fig. S4 for monoculture dynamics in patchy landscapes for each species). Some cells keep traveling with the wave from patch to corridor (*sensu* [15]) while others leave the pack to recruit and localize. This spatio-temporal pattern is a phenotype which bacteria regulate endogenously. Cells not traveling in waves can be both motile or aggregate at multiple scales [24] into non-motile clusters which are highly dynamic. Contrary to the work of Livingston and collaborators [12], which studied co-cultures of *P. aeruginosa* strains in well-mixed well plates and where dispersal was imposed in an exogenous fashion by cross-inoculation, here two species of bacteria are free to exhibit a wave-based range expansion. Can we still, in agreement with [12] and contrary to what we see in isolated well-mixed environments without cross-inoculation (Fig. 1E), observe coexistence?

### In spatial competition, pioneering *E. coli* colonizes first

**Fig. 2:**
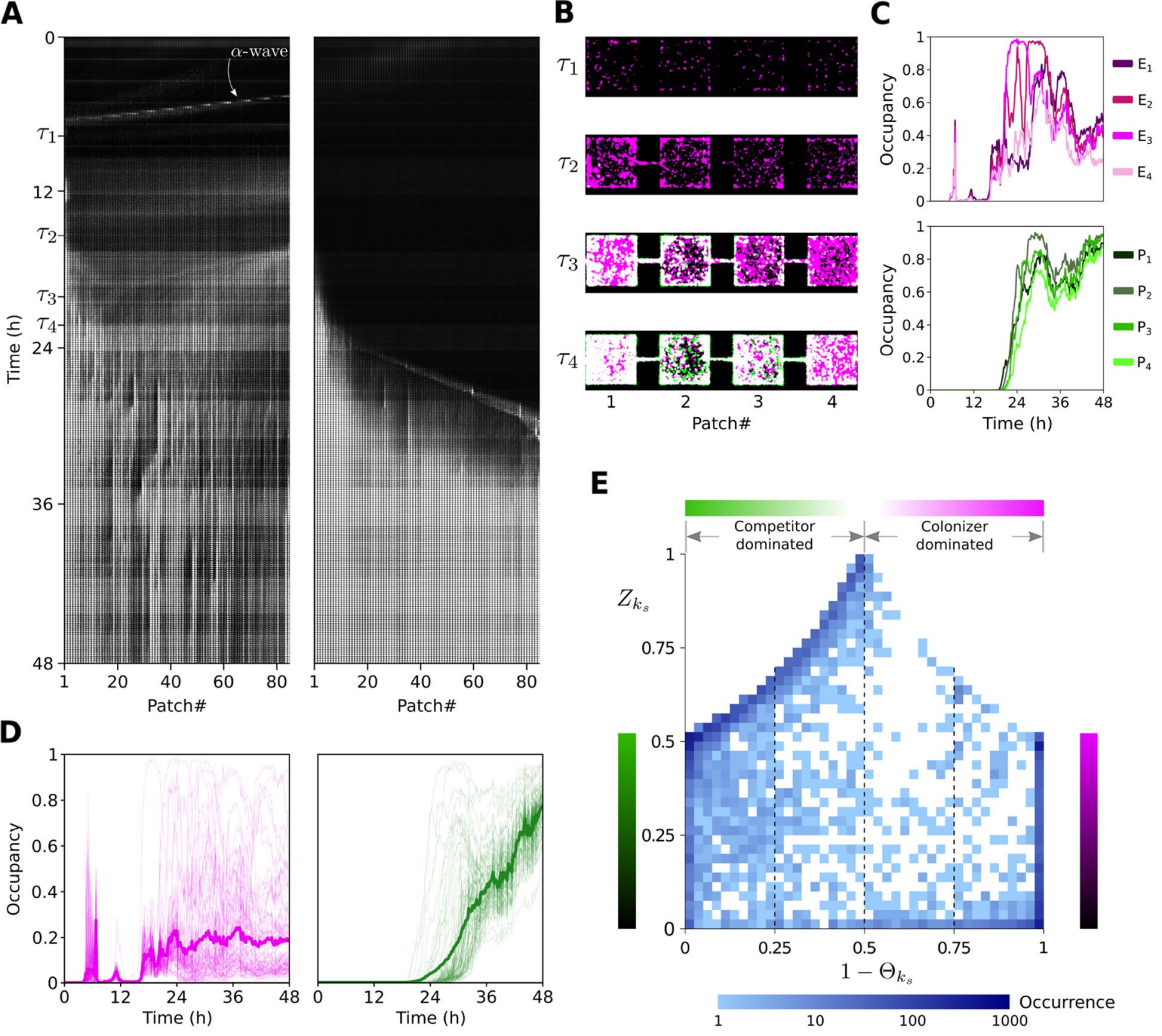
Structure and dynamics of metacommunities in patchy landscapes. **A** Kymographs built from raw fluorescence images from a competition experiment between *E. coli* (*left panel*) and *P. aeruginosa* (*right panel*). **B** Zoomed-in view of four patches $k=1\cdots 4$ at four different time points $\tau _j$ (8, 15, 20, 22 h) showing succession from *E. coli* (magenta) colonization to *P. aeruginosa* (green) invasion and eventual coexistence (white). **C** Occupancy dynamics of *E. coli*, $E_k$ (*top panel*), and *P. aeruginosa*, $P_k$ (*bottom panel*), for each of the MHPs shown in **B**. **D** Occupancy dynamics for $E_k$ (*left panel*) and $P_k$ (*right panel*) for all MHPs in the array (light curves) and spatially averaged occupancy ($\bar{E}$ in magenta, $\bar{P}$ in green) for all patches (thick curves) for *E. coli* and *P. aeruginosa* respectively. **E** Long-term local community structure after 42 hours of competition for 5100 MHPs across 60 landscapes. The vertical axis represents normalized total occupancy of MHPs $Z_{k_s}=(E_{k_s}+P_{k_s})/2$. Horizontal axis corresponds to relative fractional occupancy $(1 - \Theta _{k_s}) = E_{k_s}/(E_{k_s}+P_{k_s})$. Frequency of occurrence is depicted on logarithmic scale in blue

Following the inoculation of the patchy device inlets (left *P. aeruginosa*, right *E. coli*) initial colonization by pioneer cells of *E. coli* occurs by a low density fast traveling $\alpha$-wave *sensu* van Vliet et al. [25] (Fig. 2A, left panel). Passing through the landscape this $\alpha$-wave leaves a low density population spanning the first four patches ($k=1\cdots 4$) at $\tau _1 = 8$ hours. In Fig. 2B we show a zoomed-in view for four time points $\tau _j$. By $\tau _2 = 15$ hours division and recruitment have increased *E. coli* density reaching 35% of local occupancy (Fig. 2C, *top panel*). At this point, the first *P. aeruginosa* cells enter patches 1 and 2 (Fig. 2A, right panel). Shortly after ($\tau _3 = 20$ h), its local occupancy $P_1(\tau _3)$ begins to increase (Fig. 2C, *bottom panel*). At this stage, cells can be planktonic (free swimming) or sessile (aggregated). Subsequently, the aggregation phase progresses towards its climax; a MHP full with biofilm [14].

Aggregation events are crucial as they enhance localization of the superior competitor diminishing the connectivity of the landscape up to the point of complete fragmentation in some cases. As we show in Fig. 2B, at time $\tau _3$ hours *P. aeruginosa* has successfully invaded the first patch where it coexists with *E. coli* at high local occupancy ($P_1(\tau _3)\approx$ 50%). Due to the aggregation event of *E. coli* visible by $\tau _2$ at the corridor between patches $k=1$ and $k=2$, *P. aeruginosa* occupancy is limited dramatically ($P_k(\tau _3)$ for $k=2\cdots 4$). Eventually *P. aeruginosa* manages to penetrate the corridor and invade at time $\tau _4 = 22$ hours when all four patches host a population with significant occupancy ($P_k(\tau _4) > 0.3; k=1\cdots 4$). After 36 hours *P. aeruginosa* dominates the landscape (Fig. 2D) with local occupancy around 70% compared to 30% reached by *E. coli* (Fig. 2C). Results obtained from all patchy landscapes ($N=72$) give rise to an ensemble average showing that statistically *P. aeruginosa* coexist with *E. coli* in the long term ($\Theta \rightarrow 0.65$; Fig. 1F). While, in well-mixed experiments (Fig. 1E) *P. aeruginosa* always wins ($\theta \rightarrow 1$) after 36 h, when starting from 1:1, 10:1, 1:10 *P. aeruginosa* to *E. coli* ratios (Additional file 1: Fig. S2B).

### *P. aeruginosa* invades later but outcompetes *E. coli* locally

**Fig. 3:**
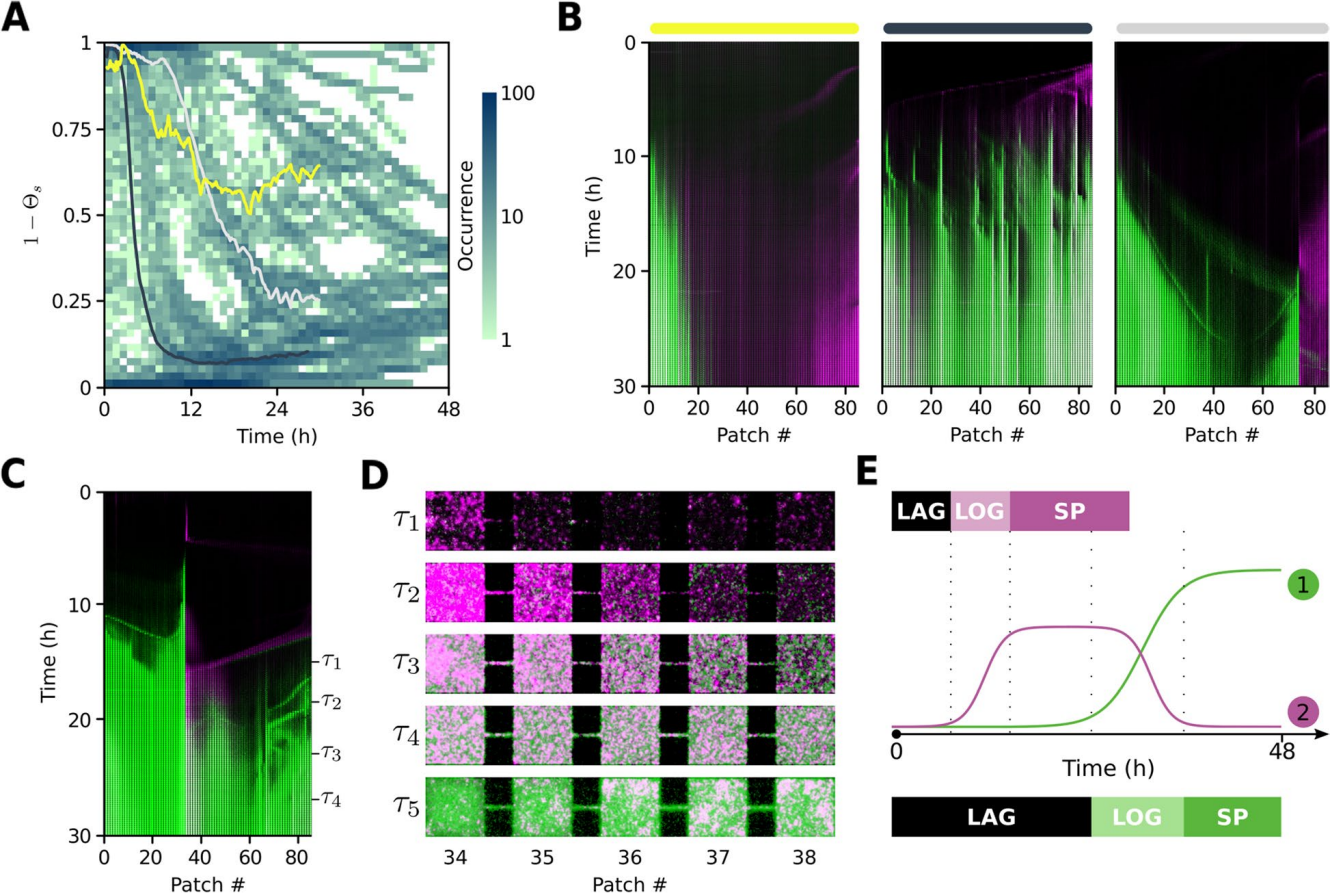
Succession, priority effects, and historical contingency. **A** Metacommunity dynamics as fractional occupancy of *E. coli* averaged over all MHPs, $(1-\Theta _s) = \bar{E}_s/(\bar{E}_s+\bar{P}_s)$, for each ($N=72$) patchy landscape, *s*, shown as a statistical ensemble. Color bar represents logarithmic occurrences of configurations for each hour. **B** Kymographs corresponding to three landscapes $s_1,s_2,s_3$ represented as three color curves (yellow, dark gray, light gray) overlayed in **A**. **C** Colonization of a patchy landscape by *E. coli* and *P. aeruginosa* for 30h of a 48h experiment. **D** Zoom-in into five MHPs of the landscape shown in **C**, over five times steps $\tau _j$ (Additional file 1: Fig. S5A for $\tau _5$). **E** Ecological succession. A fast colonizer (magenta) is replaced by a superior competitor (green) following shifted growth phase (see Additional file 1: Table S2) dynamics

After initial colonization by *E. coli*, we see a clear pattern of ecological succession, where *P. aeruginosa* enters the habitat as a densely-packed front which advances and disperses through the patchy landscape (Fig. 2A). *P. aeruginosa* occupancy ($P_k(t)$; Fig. 2D, *right panel*) demonstrates a textbook example of Skellam’s “Malthusian population in a linear habitat” [34]. This is considerably different to the fugitive dynamics (*sensu* [26]) of *E. coli* occupancy ($E_k(t)$; Fig. 2D, *left panel*) as can be appreciated in Fig. 2D. Here, we compare local occupancy $P_k(t)$ and $E_k(t)$ for all patches ($k=1\cdots 85$), as well as their landscape averages ($\bar{E}(t) =(1/85)\sum _k E_k(t)$ and $\bar{P}(t) =(1/85)\sum _k P_k(t)$; thick color curves in the foreground) before and after $t=15$ h. As *P. aeruginosa* advances, *E. coli* retreats its range of high occupancy while keeping less localized and less dense fluctuating sub-populations. These local community dynamics (Fig. 2B) are observed repeatedly, following a classical ecological succession depicted in Fig. 3E, where local species composition flows through the following states: (*i*) *E. coli* early colonization, (*ii*) expansion by recruitment, (*iii*) competition with invading *P. aeruginosa*, and eventually (*iv*) replacement by *P. aeruginosa*.

Considering all patches ($\forall k_s$) in all landscapes (indexed by *s*), the long-term pattern of local community structure can be appreciated. In Fig. 2E, we show that, in the long term, the most frequent ($\approx 1000$ occurrences) states observed fall within three classes: (*i*) fully occupied patches 100% dominated by *P. aeruginosa*, i.e., succession climax, point (0, 0.5); (*ii*) fully occupied patches 100% dominated by *E. coli*, point (1, 0.5); and (*iii*) extremely low density patches 100% dominated by *E. coli*, point $(1,\epsilon \rightarrow 0)$. Intermediate occurrences ($\approx 100$) correspond to patches with high levels of occupancy, $Z_{k_s}>0.5$, that are mostly dominated by *P. aeruginosa*, $(1 - \Theta _{k_s})\le 0.5$, or to patches highly dominated by *E. coli*, $(1 - \Theta _{k_s}) \rightarrow 1$, showing all levels of occupancy, $0.5> Z_{k_s} > \epsilon \rightarrow 0$. All other states are rare with less than $\approx 10$ occurrences among 5100 sampled MHPs. To understand this long-term statistical pattern further, we look at the transient dynamics.

In Fig. 3A, for each landscape *s*, we plot *E. coli* fractional occupancy averaged over the whole landscape as a function of time binned hourly. For each binned time point $\tau '_j$ plotted, there are $\sim 400$ landscape configurations, with a total of $\sim 19,000$ configurations over 48 hours. Early on ($\tau \le 6$ h), most occurrences ($\approx 100$) correspond to early colonization by *E. coli*, $(1-\Theta _s) = 1$. The second level of occurrences ($\approx 80$) within this early period corresponds to the initial incursion of *P. aeruginosa* which is delayed respect to *E. coli* but which nevertheless later dominates, $(1-\Theta _s) = 0$. Immediately after ($6 < \tau \le 12$), we see that the peak of occurrences ($\approx 100$) has shifted from being dominated by *E. coli* to now being dominated by *P. aeruginosa*. If we consider how occurrences distribute over the plot, we can distinguish clear trajectories of replacement. During the final phase ($24 < \tau \le 48$), most (65%) landscapes are dominated by *P. aeruginosa*. The three curves overlayed on Fig. 3A represent three landscapes shown as kymographs in Fig. 3B. Such paths correspond to: (*i*) a case where *E. coli* dominates, $(1-\Theta _{{s}_1}) > 0.5$, due to fragmentation of the spatial distribution of *P. aeruginosa*; (*ii*) a *P. aeruginosa* dominated case where *E. coli* is mostly excluded, only coexisting in low numbers, $(1-\Theta _{{s}_2}) \rightarrow 0$; and (*iii*) a case where *E. coli* persists at significant levels, $(1-\Theta _{{s}_3}) \rightarrow 0.25$.

In Fig. 3C (and Additional file 1: Fig. S5A,B), we show a kymograph where competitive pressure by *P. aeruginosa* affects the dynamics of *E. coli*. Between $12<t<18$ h, a wave of *P. aeruginosa* “chases” a wave of *E. coli*. At time $\tau _1$ *E. coli* gets trapped at patch $k=34$ encountering a ’wall’ of *P. aeruginosa* in patch $k=33$ leading to succession (Fig. 3D).

As in all experiments, what is reproduced here is the general pattern of *E. coli* colonizing first followed by *P. aeruginosa* out-competing *E. coli* locally and sometimes globally. Coexistence of alternative local community states can be observed even in adjacent patches as a consequence of stochasticity. In most experiments however, there is remarkable determinism, which emerges as the statistical pattern we can appreciate in Fig. 3A when considering the temporal ordering of events of high occurrences ($\approx 100$). At multiple scales we see the heuristic pattern of ecological succession depicted in Fig. 3E.

### Aggregation-induced fragmentation promotes coexistence

**Fig. 4:**
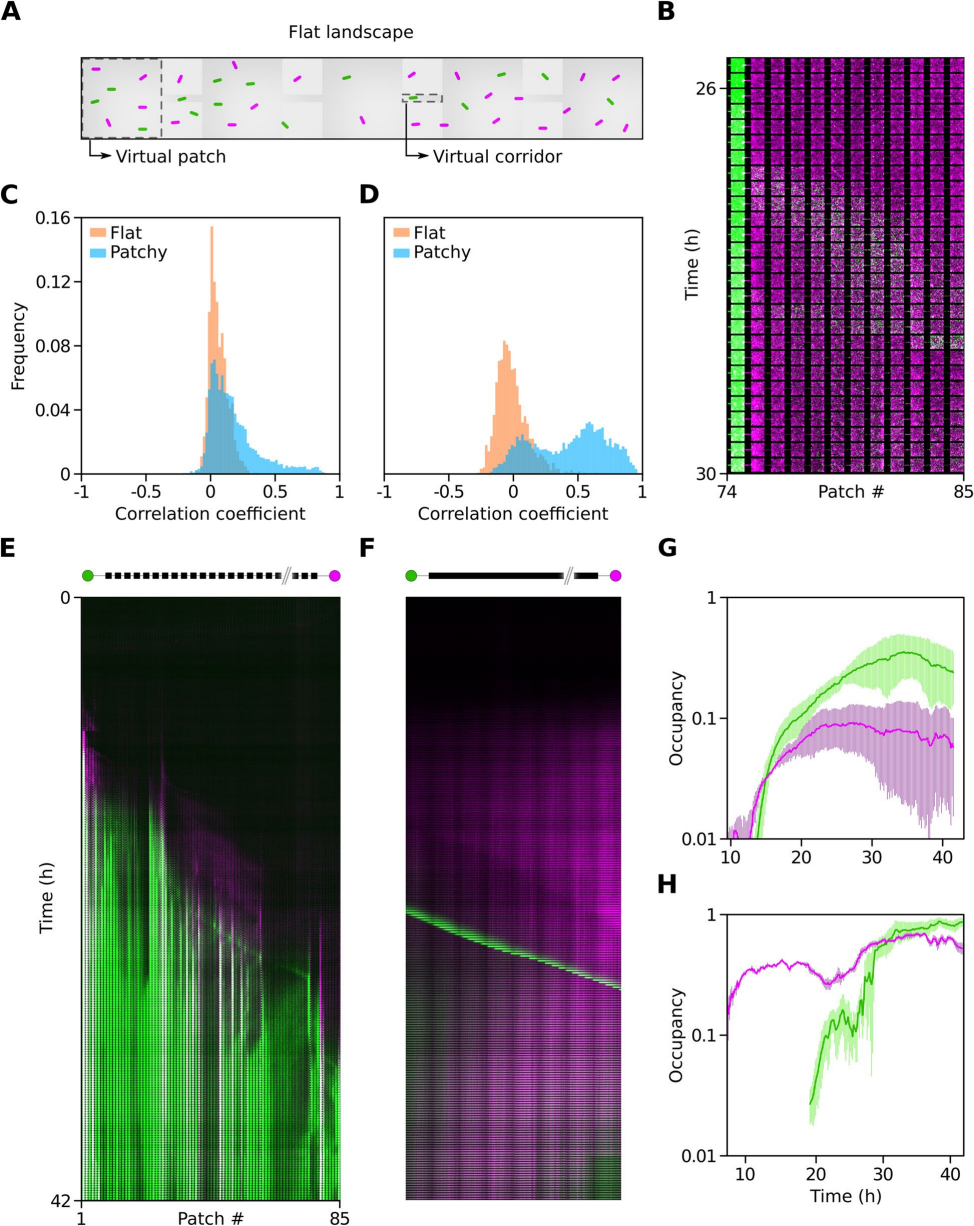
Community interactions in patchy and flat landscapes. **A** Schematic of the flat landscape with virtual patches and corridors (dashed lines) masked on top. **B** Zoom-in of patches 74-85 from Fig. 3B depicting two *P. aeruginosa* wave emissions into *E. coli* territory. **C** Distribution of Pearson coefficient between *E. coli* and *P. aeruginosa* pixel occupancy computed within virtual patches ($n = 1530$) in flat landscapes (orange) and real patches ($n = 6120$) in patchy landscapes (blue). **D** Distribution of Pearson coefficient as in **C** but for virtual (orange) and real (blue) corridor data in flat and patchy landscapes, respectively. **E** Kymograph of patchy landscape and **F** flat landscape from the same original cultures. **G**, **H** Semi-log plot of temporal dynamics of patchy (**G**) and flat (**H**) landscape occupancy for *E. coli* (magenta) and *P. aeruginosa* (green)

An important driving force for coexistence in patchy landscapes is habitat modification by *E. coli* aggregating in corridors which can disrupt later arrivals. Fragmentation events act as priority effects changing the course of succession (Fig. 3A, B). In some instances it is a momentary delay in the expansion of *P. aeruginosa*, in other cases it offers *E. coli* significant extra time and space (gap structure; Additional file 1: Fig. S5C-E) isolated from competition. An example of this can be seen in the left most kymograph of Fig. 3B where *E. coli* dominates, $(1-\Theta _{s_1}) > 0.5$. Micro-colonies in the corridors have a jamming effect, illustrated in Fig. 1B, where competitive interactions are localized. In extreme cases this turns into fragmentation and the superior competitor is unable to overcome this barrier. The barrier is sometimes broken by de novo waves which can penetrate the competitor’s territory. Such an event can be seen in the right most kymograph in Fig. 3B. Here, a wave of *P. aeruginosa* collides at time $t = 20$ h with *E. coli* aggregated at the corridor located between patches $k=74$ and $k=75$. This interaction can be appreciated post-collision in Fig. 4B. The wave of the superior competitor (green), is first unable to penetrate the territory held by *E. coli* (magenta) and reflects back as well as diffracting (*sensu* [25]), giving rise to a *P. aeruginosa* sub-population localized at MHP number 74. From this sub-population *P. aeruginosa* periodically emits new waves of subsequently higher cell numbers which eventually percolate through the barrier. Such wave invasions into hostile territory are reminiscent of *E. coli* expansion into landscape ecotopes with high concentration of antibiotics [35] and highlights the importance of waves for bacterial fitness.

To test if patchiness facilitates priority effects, we ran experiments in “flat” non-patchy landscapes and compared them to experiments in patchy ones which were started from the same cultures ($n = 3$). Coexistence was also observed in flat landscapes despite the fact that *E. coli* micro-colonies are unable to fragment the landscape. Here, coexistence is due to the emergence of a neutral ecology where species reach a similarly high level of long-term ($t^* \gg 30$ h) average occupancy, $\langle \bar{E}(t^*)\rangle _{\textrm{flat}} \approx \langle \bar{P}(t^*)\rangle _{\textrm{flat}}$ (Fig. 4H). In contrast, in the patchy system we see significantly different levels of long-term average occupancy, $\langle \bar{E}(t^*)\rangle _{\textrm{patchy}} \ll \langle \bar{P}(t^*)\rangle _{\textrm{patchy}}$ (Fig. 4G).

In Fig. 4E, we show a *P. aeruginosa* (green) expansion front invading a patchy landscape and displacing *E. coli* (magenta) contrasting the result for a flat landscape (Fig. 4F), where we see a fast traveling wave of *P. aeruginosa* in coexistence with *E. coli*. This wave does not leave localized sub-populations behind as is the case for waves in patchy landscapes (Fig. 4E), but a rather homogeneous distribution of cells which after 36 hours show only minor long-term averaged occupancy differences (Fig. 4H). Contrary to patchy landscapes, the flat landscape has no intermediate patch scale and produces no separation between local population growth and landscape-scale range expansion.

From additional flat landscape experiments (6 devices, 18 landscapes), a statistical comparison was made regarding the spatial correlations between *P. aeruginosa* and *E. coli* using Pearson coefficient. For patchy landscapes, data was partitioned between patches and corridors for all 72 landscapes, thus rendering $n = 6120$ pairs of both *P. aeruginosa* and *E. coli* pixel occupancy for each ecotope. Similarly, for flat landscape data, 85 virtual patch and corridor masks were generated *sensu* [36] rendering $n = 1530$ replicates, see Fig. 4A.

When comparing virtual and real patches in the flat and patchy landscapes, respectively, we observe a near neutral, but slightly positive correlation describing average interactions (Fig. 4C). While virtual patches show less variance and no significant skew, the real patch distribution demonstrates a clear positive skewness. This suggests a disproportionate number of competing—co-localized— cells in patchy landscapes compared to flat landscapes.

Comparing the distributions of virtual and real corridors, differences become qualitative (Fig. 4D). For virtual corridors we see no significant correlation response with a slightly larger variance than that found for the virtual patch distribution. This is expected, as the smaller region masked by the virtual corridor permits more stochasticity. Remarkably, we found a bi-modal distribution for real corridors. The second peak emphasizes the significance of interactions at corridors, highlighted by the different dynamics (Fig. 4E, F). Analysis of micro-colony sizes in patchy versus flat landscapes provides further evidence for the relaxation of the competitive hierarchy in flat landscapes (Additional file 1: Fig. S6).

### A spatial model of the CC trade-off

The competition-colonization (CC) trade-off [37] has been proposed to explain coexistence in a number of diverse ecosystems, from the intertidal zone [38] to grasslands [39] and coral reefs [40]. Inspired by our empirical observations, we developed a spatially-explicit stochastic model. We incorporate a localized mixed state where both species can occupy a common site at the same time with the condition they are locked into an interference competition program. While locked in this state, no dispersal takes place, only competitive lottery.

**Fig. 5:**
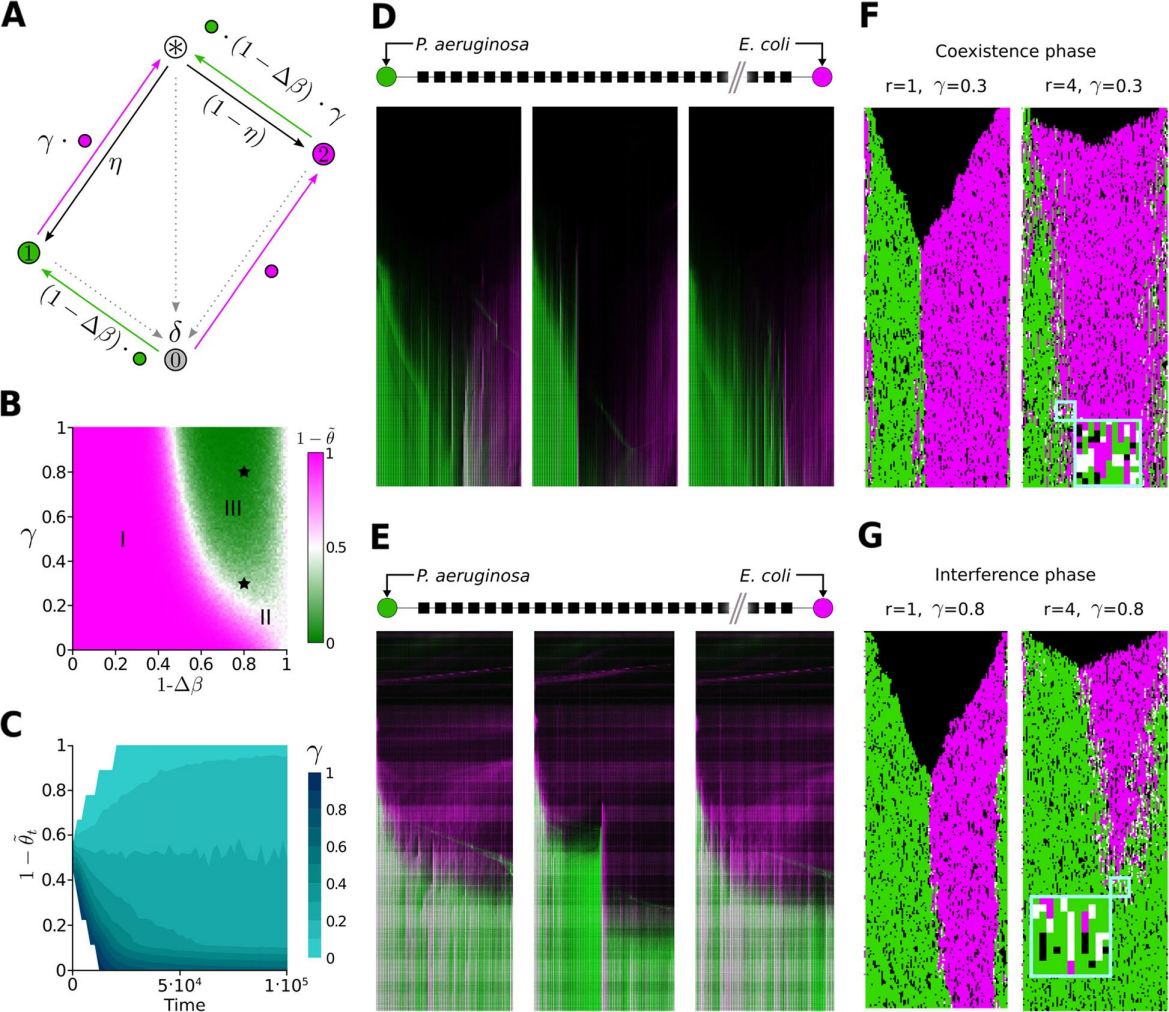
Insights from a stochastic spatial model. **A** Markov chain describing the transitions of our model; colonization rates are dependent on the state of neighboring sites. **B** Long-term behavior of our model with nearest neighbor ($r=1$) interaction. Three phases as a function of $(1-\Delta \beta )\times \gamma$ depict differences in colonization $\Delta \beta$ and priority effects $\gamma$. Average colonizer fractional abundance $(1-\tilde{\theta }) =\langle \rho _E \rangle /(\langle \rho _E \rangle +\langle \rho _P \rangle )$ is shown as magenta-to-green color map. Here, $\Delta \beta = \beta _1-\beta _2$, $\alpha =4$, $\beta _1\equiv 1$ and $\delta =0.1$. **C** For a fixed value of colonization difference, $(1-\Delta \beta )=0.8$, metacommunity dynamics, $(1-\tilde{\theta }_t)=\rho _E(t)/(\rho _E(t)+\rho _P(t))$, are shown for different values of $\gamma$ (cyan gradient bar) with other parameter values same as in **B**. **D**, **E** Three landscapes where we observe regimes of coexistence (**D**) or dominance (**E**) behavior. **F**, **G** 1D spatial transects of 2D simulations with parameter values shown as stars in the phase space shown in (B) ($r=1$; left) and for higher interaction range ($r=4$; right). Simulations correspond to *coexistence* phase (II) for $\gamma =0.3$ (**F**) and *interference* phase (III) for $\gamma =0.8$ (**G**). Both scenarios $(1-\Delta \beta )=0.8$

We consider sites on a lattice representing a local community: vacant (state 0), occupied by competitor (state 1), colonizer (state 2), or both (mixed state $*$). State transitions are sketched in Fig. 5A where interactions occur in a local neighborhood (See 5 and Additional file 2: Supplementary Notes 2 and 3). We performed numerical studies and explored the parameter space $[(1-\Delta \beta ) \times \gamma ]$ representing differences in colonization ability $(1-\Delta \beta )$ and priority effects $\gamma$ (Fig. 5 and Additional file 1: Fig. S7).

In Fig. 5B, we show our model’s long-term colonizer fractional occupancy $(1-\tilde{\theta })$ with nearest neighbor interactions. We recognize three phases: (I) a *scramble* phase, where only the colonizer survives; (II) a *coexistence* phase, where both persist; and (III) an *interference* phase, where the competitor wins. In Fig. 5, we show three patchy landscapes congruent with the *coexistence* (Fig. 5D) and *interference* (Fig. 5E) phases of our model. Fixing the value representing colonization differences, $(1-\Delta \beta )= 0.8$, we can think of individual experiments as different scenarios of priority effects changing stochastically. Using a value $\alpha =4$ with a 20% difference ($\Delta \beta _0=0.2$) in dispersal ability (70% chances of losing interference competition; $\eta (0.2)=0.698$), in Fig. 5C, we show the average dynamics of colonizer fractional occupancy for different scenarios of priority effects (see Additional file 1: Fig. S7C-F for other values of $\alpha$). Notice the importance of parameter $\gamma$ in determining trajectories of competitive replacement as well as long-term patterns of dominance (phases). For a 20% decrease in cross-colonization rate due to priority effects ($\gamma = 0.8$), we obtain a scenario within the *interference* phase (Fig. 5G), and the competitor pushes the colonizer off the landscape (Fig. 5E). Decreasing $\gamma$ further results in another scenario we observed in experiments (Fig. 5D); *E. coli* persists due to the blockage of patch-corridor interfaces. Such scenario occurs for parameter $\gamma = 0.3$ where simulations lay within the *coexistence* phase (Fig. 5F). A stochastic process $\gamma _t$ would account for trajectories like the ones in Fig. 3A.

For no priority effects ($$\gamma =1$$), we distinguish two critical values $$\Delta \beta ^*$$ and $$\Delta \beta ^{**}$$ delimiting the three phases at the top of Fig. 5B: (i) If the difference in colonization ability is large, $$1> \Delta \beta >\Delta \beta ^*$$, the system is in the *scramble* phase; (ii) if the difference is of intermediate magnitude, $$\Delta \beta ^*> \Delta \beta > \Delta \beta ^{**}$$, the system is in the *coexistence* phase; and (iii) if small differences are considered, $$\Delta \beta ^{**}>\Delta \beta \ge 0$$, the system is in the *interference* phase. When no differences exist, $$\Delta \beta =0$$, there is *neutral* coexistence as the lottery has no bias ($$\eta =0.5$$) regardless of the trade-off strength $$\alpha$$. The nearly neutral case ($$\Delta \beta \cong \epsilon \ne 0 : \epsilon \rightarrow 0$$) allows us to appreciate the role of $$\gamma$$ which induces fragmentation and leads to an extinction threshold at $$\gamma _c$$.

Scanning along a transect $(1-\Delta \beta _0) = 0.8$ across the phase space varying $\gamma$ from no priority effects ($\gamma =1$) to full cross colonization inhibition ($\gamma =0$) in Fig. 5B, we traverse two transitions between phases: (i) a boundary between *interference* and *coexistence* and (ii) *coexistence* and *scramble*. Thus, at intermediate values of priority effects, we can expect coexistence for a pair of strategies with a small difference in colonization, $0<\Delta \beta _0 < 0.3$. The sequestration from colonization, produced by localization of the interference interaction, has a critical role in modulating coexistence. This result is congruent with our finding that *E. coli* coexist with *P. aeruginosa* by a localization and fragmentation mechanism, thus highlighting the importance of priority effects occurring at the corridors.

## Discussion

Combining theory and experiments, we demonstrate that, in a synthetic two-species community of motile strains, *E. coli* is a fugitive species while *P. aeruginosa* is a superior competitor enacting a competition-colonization trade-off in patchy environments. Aggregation-dependent priority effects deterring competitor dispersal provide a mechanistic explanation for coexistence and a textbook example of niche pre-emption [17].

In patchy environments, bacterial life history resonates with periodic habitat structures [31, 32] and selects patterns of localization which reflect a balance between exploiting patches by entering stationary phase programs or exploring the landscape for new colonization opportunities. In flat landscapes of approximately equivalent volume but lacking such periodic structure, a neutral ecology emerges (Fig. 4). Microbial metacommunities are not only composed by localized sub-populations but also contain traveling waves. Waves generate dispersal structures powering ecological succession at the scale of patches (Figs. 2B and 3D) and fugitive dynamics at the landscape level (Fig. 2D). Initial regional species pool conditions, their initial co-occurrence and distribution also influence the effectiveness of wave propagation as a method for fugitive strategies to coexist with superior competitors (see Additional file 1: Fig. S3 and Additional file 2: Supplementary Note 1). Flat landscapes offer no corridor-to-patch transitions, therefore inducing a different ecology (neutral versus competition-colonization) and pattern of micro-colony structure (Additional file 1: Fig. S6). Together, these two landscape types allowed us to study the role patchiness plays in structuring a community of two bacterial species. Although each individual experiment in patchy landscapes deviated from the heuristic pattern depicted in Fig. 3E in a multi-scale stochastic fashion, an ensemble average can be constructed for dynamics (Figs. 1F; 3A) and long-term structure (Fig. 2E).

A mechanism for coexistence in patchy landscapes is habitat fragmentation by blockage of patch-corridor interfaces (Figs. 1B; 2B; and 4B,D). These priority effects are responsible for holding back *P. aeruginosa* which in some cases ultimately can become fragmented. From the perspective of succession, coexistence is transient as waves of competitors will eventually break through (Fig. 4B). Corridors are ecotopes where strong interactions take place (Fig. 4D); by delaying *P. aeruginosa*’s arrival, *E. coli* increases its window of opportunity. The longer *E. coli* has free of competition, the more it increases its chances against *P. aeruginosa* and sometimes can dominate (Fig. 3A, B).

Extending the CC model to consider the spatial structure of scramble and interference competition explicitly and acting separately allowed us to qualitatively understand the large-scale dynamics of our experiments (Fig. 5). By varying spatial structure experimentally (flat vs. patchy landscapes), we can manipulate the location along the trade-off in which community assembly occurs (neutral vs. CC). Spatial aspects of wave mechanics such as diffraction are missing in our model and need to be developed. Nevertheless, its current formulation highlights the importance of priority effects driving patterns of competitor co-localization and fragmentation. We expect wave diffraction to be the mechanism revealing how the topology of the habitat triggers a transition from neutral ecology to succession dynamics.

Progress towards understanding the role of historical contingency in shaping community composition has enormous implications for our capacity to predict, modify, and react to the dynamics of microbiomes [41]. Similar examples of niche-preemption to those documented above have been described in the colonization process by *Bacteroides fragilis* in the human colon post antibiotic treatment [42]. Beyond the example of interference competition shown in this work, the order of species colonization can also change the trajectory of microbiome composition by way of nutrient depletion—i.e., exploitative competition. Pairwise experiments using 11 rhizobacteria species showed how composition depended on order of arrival as some species inhibited colonization of subsequent species via iron depletion [43]. As a ubiquitous phenomenon in all ecological communities, the process of succession should continue to be a focus for spatial microbial ecology studies.

## Conclusion

In conclusion, our work shows that when habitats are patchy there is a CC trade-off and ecological succession is to be expected, highlighting the importance of dispersal in structuring microbiomes. For multi-species systems, this trade-off provides a method to decompose the dynamics based on positioning life history strategies along this spectrum of differentiation.

## Methods

### Strains and growth conditions

Experiments were performed using wild-type strains of *E. coli* (JEK1036 and JEK1037) and *P. aeruginosa* (PUPa3-G and PUPa3-R) labeled with green and red fluorescent proteins (GFP and RFP). Strain details are listed in Additional file 1: Table S3. For both species, strains are isogenic except for fluorescence labeling.

Single colonies for both *E.coli* and *P.aeruginosa* were grown from $-80^{\circ }\textrm{C}$ glycerol stocks on solid LB agar plates (LB Broth EZMix, Sigma-Aldrich + 1.5% Bacto Agar, MOLAR Chemicals) and subsequently inoculated in 3mL Lysogeny Broth medium (LB Broth EZMix, Sigma-Aldrich) for 16h ± 30min overnight at $30^{\circ }\textrm{C}$, $200 \textrm{rpm}$. For *P. aeruginosa*, $50\mu \textrm{M}$ of Ampicillin (Amp) and Gentamicin (Gm) was added. Overnight cultures of *E. coli* (*P. aeruginosa*) were back-diluted 1:1000 (1:500) in $3\textrm{mL}$ LB medium with $1\textrm{mM}$ Isopropyl $\beta$-d-1-thiogalactopyranoside, IPTG, (50$\mu$g/mL Amp and Gm) and grown to an optical density at $600\textrm{nm}$ (OD$_{600}$) of 0.3. Cultures were then centrifuged at $350\textrm{G}$ for 10 min, after which the supernatant was removed and cells were resuspended in LB medium containing $1\textrm{mM}$ IPTG . PUPa3-R and PUPa3-G maintain their plasmids over the course of the experiment (48 h) in absence of antibiotics Amp and Gm in the growth media. Stability of the plasmid was confirmed by comparing cell number in bright field and fluorescence images over a period of 3 days (results not shown).

### Well-plate assays

#### Monoculture growth curves

Cultures of JEK1037/36 and PUPa3-G/R were grown overnight until stationary phase and back-diluted 1:1000. Once back-diluted and cultures had reached an $\textrm{OD}_{600} = 0.3$, they were again back-diluted 1:100 into a 96 well-plate well reaching a final volume of $100\mu$L. Monoculture growth patterns were evaluated in 96 well-plates using fresh LB. Plates were incubated in a Synergy two microplate reader at 30 $^{\circ }\textrm{C}$ with continuous shaking; $\textrm{OD}_{600}$ measurements were acquired every 10 min for a total of 48 h. A total of 6 replicates were used for each of the 4 strains shown in Fig. 1C.

#### Co-culture—monoculture comparison

JEK1037 and PUPa3-G strains were grown overnight until stationary phase ($30^{\circ }\textrm{C}$, 200 rpm) in LB medium supplemented with IPTG (for *E. coli*) or antibiotics (50 $\mu$g/mL Amp and Gm for *P. aeruginosa*). Overnight cultures were back-diluted 1:1000, incubated to grow until reaching $\textrm{OD}_{600} = 0.3$ after which centrifuged twice (4000 rpm for 5 min) to then be re-suspended in fresh LB without antibiotics. A 1:100 dilution was applied into a 96 well-plate well for each monoculture reaching a final volume of 100$\mu$L. Co-cultures were prepared by adding the two strains in 1:1 ratio into the wells. The plate was incubated in a Synergy H1 microplate reader at $30^{\circ }\textrm{C}$ with continuous shaking. Measurements of $\textrm{OD}_{600}$ were performed every 5 min for a total of 24 h. From three mono- and three co-cultures, three colony-forming unit (CFU) assays were performed for each culture; 50$\mu$L of 1:$10^5$ and 1:$10^6$ dilutions of each culture were homogeneously spread on agar plates, which were incubated at room temperature for 1.5 days. For the mixed cultures, *E. coli* and *P. aeruginosa* colonies can easily be distinguished based on colony morphology. Results are shown in Additional file 1: Fig. S1.

### Well-mixed competition experiments

Cultures of JEK1037 and PUPa3-G were grown overnight until stationary phase and back-diluted 1:10000 in $25\textrm{mL}$ of fresh LB and 1mM IPTG grown in $150\textrm{mL}$ Erlenmeyer flasks resulting in a 1:1 initial cell density *E. coli*:*P. aeruginosa*. Asymmetric initial conditions (10:1 and 1:10) were also tested on the same day. Each experimental set-up was performed with 3 replicates. Flasks were placed in a shaking incubator set at 30$^{\circ }\textrm{C}$ and $100\textrm{rpm}$ for a total of 48 h. Optical density measurements ($\textrm{OD}_{600}$) were taken every 12 h in parallel with cell counting measurements, for which $1 \textrm{mL}$ sample cultures were diluted to $\textrm{OD}_{600}=0.4$ and $1 \mu \textrm{l}$ droplets were placed on $1.1\textrm{mm}$ thick agar pads. Droplets were allowed 5 min to absorb to the agar pads before placing a cover-slip on top to ensure an even distribution of cells in a symmetric, circular region of interest (ROI). Images were acquired in the center of this ROI using a 40X Nikon Plan Fluor objective giving a field of view of $(413\times 348.4\mu \textrm{m}^2)$. Average number of *E. coli* ($\bar{N}_E$) and *P. aeruginosa* ($\bar{N}_P$) cells in the ROI were counted using ImageJ [44].

### Microfluidic device fabrication and preparation

Microfabricated devices consist of two inlet holes ($1.2\textrm{mm}$) on opposite sides with 3 parallel landscapes, each with an inlet strip leading into arrays of 85 habitat patches ($100\times 100\times 5\mu \textrm{m}^3$) connected by corridors ($50\times 5\times 5\mu \textrm{m}^3$) (Fig. 1A and Additional file 1: Fig. S2) or one long quasi-2D environment ($12750\times 100\times 5\mu \textrm{m}^3$). In the vertical dimension, a maximum of 2–3 cells can stack. Devices were fabricated using soft lithography techniques [29]: a silicon wafer was coated with a thin film (5$\mu$m, height of the device) of the negative photoresist SU-8 (SU-8 2005, MicroChem), and the design of the device was written into the resist with a laser pattern generator ($\mu$PG 101, Heidelberg Instruments) to fabricate a master mold on which polydimethylsiloxane, PDMS (10:1 PDMS:curing agent; Sylgard 184, Dow Corning), was deposited to yield an elastomeric stamp that was covalently bonded to a glass cover slip by oxygen plasma activation (29.6 W, 400mTorr, 45 sec; PDC-002, Harrick Plasma) of both the PDMS and glass parts.

#### Microfluidic experiments

For each experiment, a device containing 3 habitat landscapes each, was inoculated with either the red PUPa3-R or green PUPa3-G strain of *P. aeruginosa* used with the green JEK1036 or red JEK1037 *E. coli* strain, respectively. This way experiments were performed with a green-red or red-green pair of *P. aeruginosa* and *E. coli*.

Prior to inoculation, the devices were wettened with LB + $1\textrm{mM}$ IPTG. Then $1\mu \textrm{l}$ culture of *P. aeruginosa* and *E. coli* was pipetted into the inlet holes on opposite ends of the device and the device was sealed with fast curing PDMS (Kwik-Sil Silicone Elastomer, World Precision Instruments). A water tight wall around the perimeter of the sealed device using four $24\times 60\textrm{mm}$ coverslips glued onto the glass-slide using fast curing PDMS was added. The sealed device was then submerged in Milli-Q water to prevent the device from drying out. During the 48 h time-lapse microscopy experiment, the microscope set-up was equipped with an incubator enclosure (Okolab) by which temperature is controlled at $30^{\circ }\textrm{C}$ during image acquisition.

Alternative initial conditions were also considered (see Additional file 1: Fig. S3 and Additional file 2: Supplementary Note 1). JEK1037 and PUPa3-G strains were grown as described in “Strains and growth conditions,” but instead of being inoculated separately on opposite ends of the device were mixed 1:1 prior to inoculation. Three inoculation protocols were performed: (*i*) flat landscape and patchy landscape—single inlet, (*ii*) patchy landscape—both inlets, (*iii*) flat landscape, and patchy landscape—distributed across the entire landscape.

### Image acquisition and data analysis

Devices were imaged at 10 minute intervals using a Nikon Eclipse Ti-E microscope equipped with 10X Nikon Plan Fluor objective, GFP and mcherry fluorescence filter sets (49002 & 49008, Chroma Inc.), Andor Neo sCMOS camera (Andor Technology plc.), and LUMEN 200 Pro metal arc lamp (Prior Scientific Ltd.). A Prior Proscan II motorized stage (Prior Scientific Ltd.) was used for scanning. The NIS Elements Ar software (Nikon Inc.) was used for image acquisition and data processing. Image analysis was carried out using ImageJ and Python. Fluorescence intensity is a poor estimation for biomass due to differences in expression among cells. To avoid this problem we use a custom script in ImageJ to convert all images to occupancy data for each of the two strains used in an experiment.

#### Local occupancy within patches (MHPs)

For every timestep, background correction is performed on each image and a threshold pixel value is calculated based off the corresponding auto-fluorescence for each of the color channels used (red and green). Above this threshold the pixel ($0.803\mu \textrm{m}^2$) is considered occupied by that strain (value of 1), otherwise it is vacant (value of 0). Using the ROImanager in ImageJ, a custom mask was fitted at each patch for each experiment allowing us to consider occupancy values at the patch level for each strain. *E. coli* (*P.aeruginosa*) occupancy for a MHP indexed by *k* is denoted as $E_k$ ($P_k$). Notice that occupancy is normalized per channel, thus $0\le E_k \le 1$ and $0 \le P_k \le 1$. Total MHP occupancy $Z_k=P_k+E_k$ and fractional occupancy of *P. aeruginosa* $\Theta _k = P_k/(E_k + P_k)$ and *E. coli* $(1-\Theta _k)= E_k/(E_k+P_k)$ was calculated from acquired occupancy data. We characterized a local MHP community at location *k* at time *t* as $\{\Theta _k, Z_k\}$ or $\{(1-\Theta _k), Z_k\}$ depending if focus is on *P. aeruginosa* or *E. coli* respectively.

While experiments are carried out with both green-red and red-green pairs of *P. aeruginosa* and *E. coli*, for all figures, images are colored green for *P. aeruginosa*, magenta for *E. coli* and white for both. This is done to facilitate easy interpretation of the results and comparison to the model presented.

### Numerical simulations

The C programming language was used to implement simulations of the stochastic spatial model using the Mersenne Twister algorithm [45] for pseudo random number generation. For plotting the spatio-temporal patterns of individual simulations (Fig. 5F, G) we used the GTK+ library. In order to obtain results shown in Fig. 5B, C, F, G; S4D-F we used a two dimensional (2D) lattice $\mathcal {L}$ composed of $100\times 100$ sites and periodic boundary conditions. As initial condition, in all simulations, the lattice was seeded with 5 sites occupied by the superior competitor (state 1) and fast colonizer (state 2) strategies each. A $100 \times 100$ sweep of the $[(1- \Delta \beta ) \times \gamma ]$ parameter space was performed. Individual colored pixels constitute long-term results of individual simulations. For each run, after converging to a statistical steady state (after $1 \cdot 10^5$ time steps) an ensemble average was then computed over the next $10^3$ time steps.

## Supplementary information

**Additional file 1:** **Figure S1.** Well-mixed experiments. **Figure S2.** Microfluidic devices. **Figure S3.** Alternative initial conditions. **Figure S4.** Monoculture experiments in patchy landscape. **Figure S5.** Structure and dynamics of a bacterial metacommunity. **Figure S6.** Micro-colony structure in patchy versus flat landscapes. **Figure S7.** Models of the competition-colonization (CC) trade-off. **Table S1.** Symbols. **Table S2.** Glossary. **Table S3.** Bacterial strains.

**Additional file 2:** **Supplementary Note 1.** Alternative initial conditions inoculating with a 1:1 mixture of species. **Supplementary Note 2.** Stochastic spatial model. **Supplementary Note 3.** Patch occupancy and the competition-colonization trade-off.

## Data Availability

The design mask used for our microfluidic devices as well as all ImageJ/Python scripts as well as code for model and data from the kymographs are deposited in the public repository Science Data Bank: (10.57760/sciencedb.06193).

## Abbreviations

CC: Competition-colonization
MHP: Microhabitat patch
LB: Lysogeny broth
GFP: Green fluorescent protein
RFP: Red fluorescent protein
Amp: Ampicillin
Gm: Gentamicin
IPTG: Isopropyl $\beta$-d-1-thiogalactopyranoside
PDMS: Polydimethylsiloxane
OD (OD$_{600}$): Optical density measured at a wavelength of 600 nanometers
CFU: Colony-forming units
ROI: Region of interest
2D: Two-dimensional
1D: One-dimensional
