# Supplementary material for: Ecological succession and the competition-colonization trade-off in microbial communities
Source: BMC Biol. 2022 Nov 30;20:262. doi: 10.1186/s12915-022-01462-5 (PMC9710175; doi:10.1186/s12915-022-01462-5)
Supplement: Supplementary file 1 — Additional file 1: Figure S1. Well-mixed experiments. Figure S2. Microfluidic devices. Figure S3. Alternative initial conditions. Figure S4. Monoculture experiments in patchy landscape. Figure S5. Structure and dynamics of a bacterial metacommunity. Figure S6. Micro-colony structure in patchy versus flat landscapes. Figure S7. Models of the competition-colonization (CC) trade-off. Table S1. Symbols. Table S2. Glossary. Table S3. Bacterial strains. [file 12915_2022_1462_MOESM1_ESM.pdf]

Ecological succession and the competition-colonization trade-off in microbial communities

Miles T. Wetherington [1,2,3], Krisztina Nagy [2], László Dér [2], Ágnes Ábrahám [2,4], Janneke Noorlag [1,6], Peter Galajda [2] and Juan E. Keymer [1,5,6]

[1]Department of Ecology, School of Biological Sciences, P. Catholic University of Chile

[2]Biological Research Centre, Institute of Biophysics

[3] School of Applied and Engineering Physics, Cornell University

[4]Doctoral School of Multidisciplinary Medical Sciences, University of Szeged

[5]Institute of Physics, School of Physics, P. Catholic University of Chile

[6]Department of Natural Sciences and Technology, University of Aysén

Additional file 1

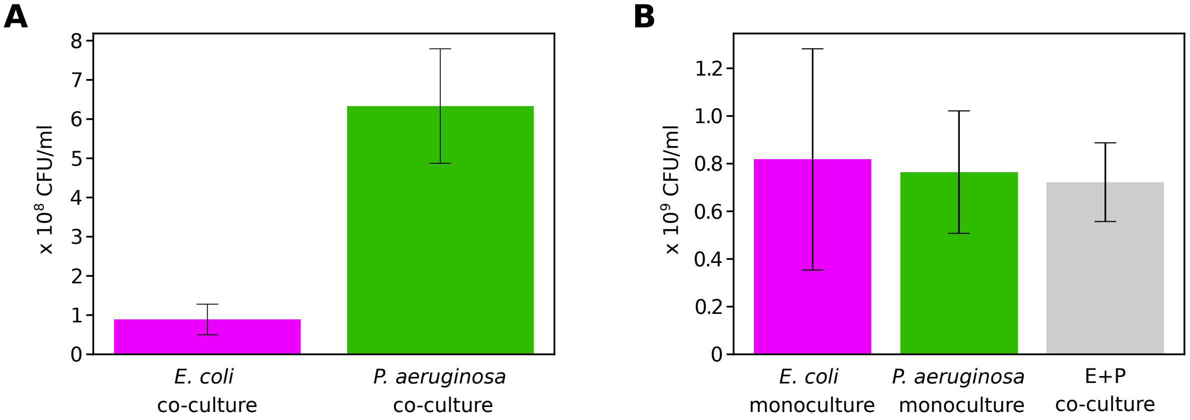

Figure S1: Well-mixed experiments Results of well-mixed co-cultures and monocultures of *E. coli* (E) and *P. aeruginosa* (P). **A** CFUs for both strains in co-culture competition experiments after 24h in well-plates. **B** Total CFUs for monocultures for each species of bacteria and for co-culture after 24h in well-plates.

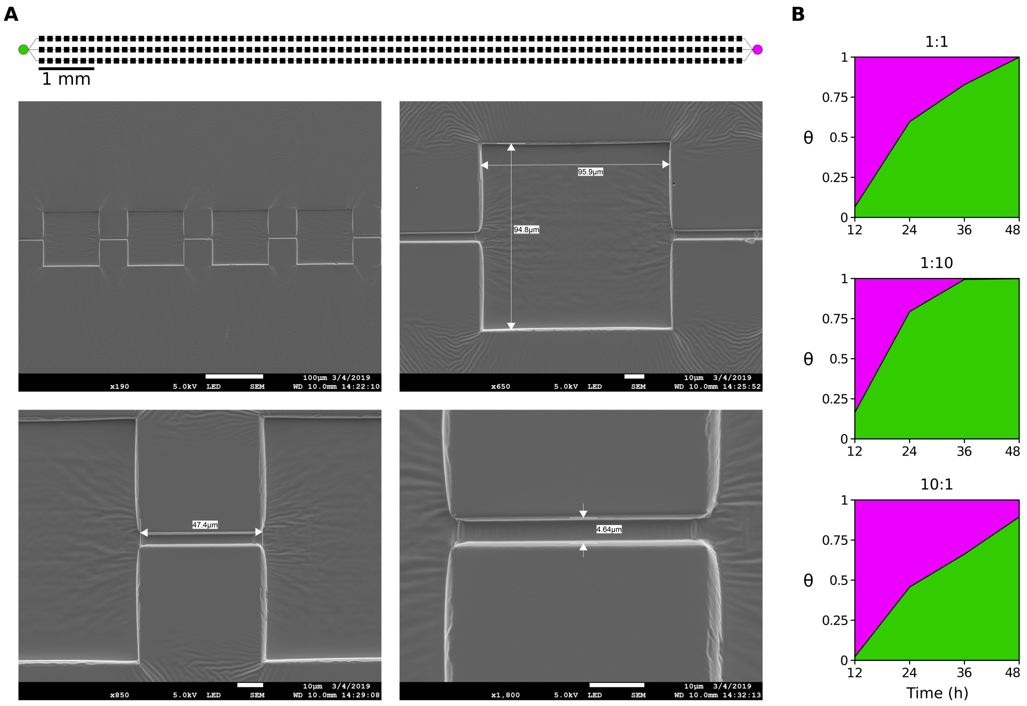

Figure S2: Microfluidic devices A Scanning electron microscopy images of an MHP array (patchy landscape) in the microfluidic devices depicted in Figure 1A. PDMS devices (see Methods) were sputter coated using a Q150T ES turbomolecular pumped coater leaving a 15nm gold film deposition and then imaged using a JSM-7100F Thermal field emission electron microscope. **B** Community dynamics in well-mixed environments represented as *P. aeruginosa* fractional occupancy, $\theta \equiv \langle N_P/(N_E + N_P)\rangle$, *P. aeruginosa* to *E. coli* initial inoculation ratios 1:1, 1:10, and 10:1.

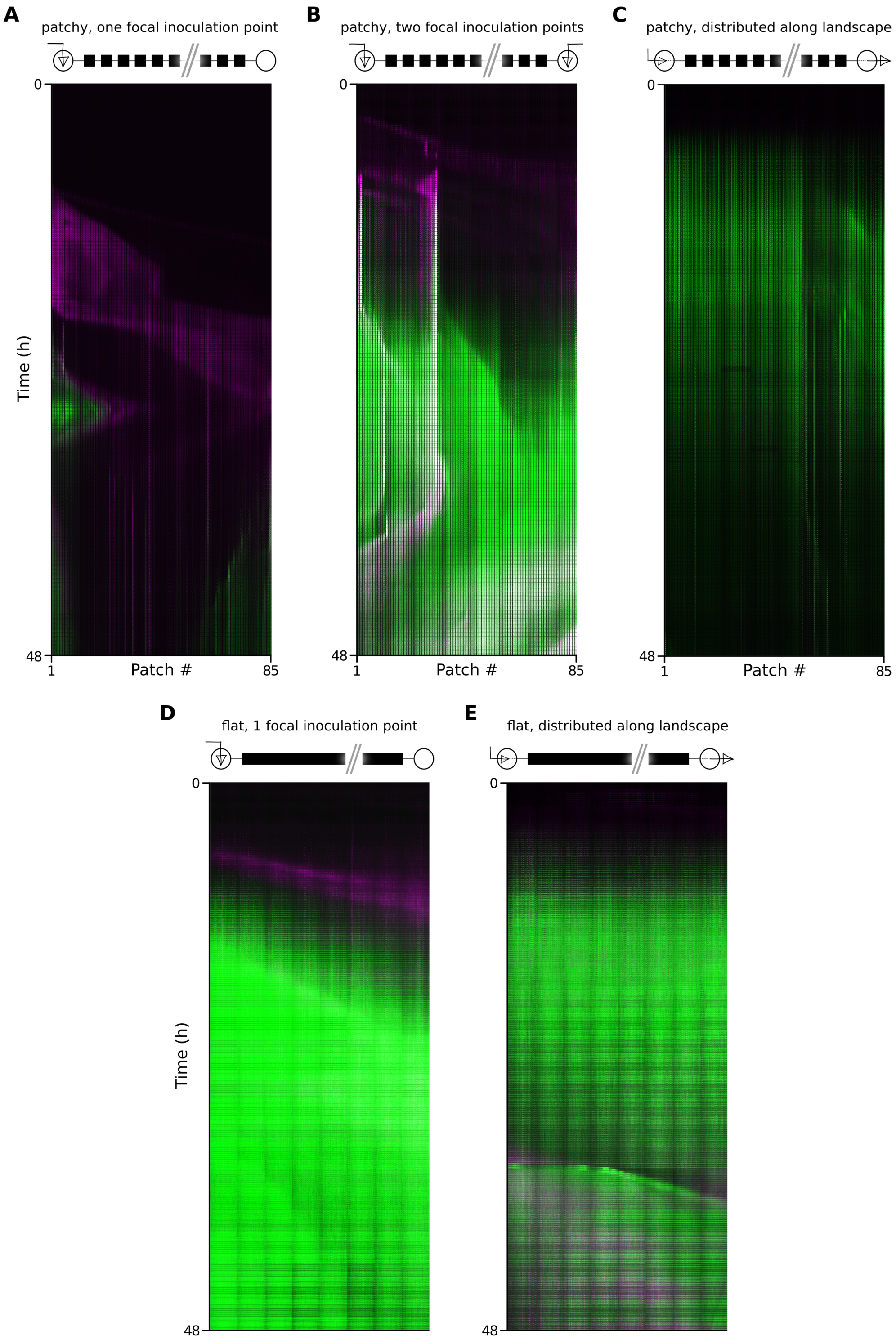

Figure S3: Alternative initial conditions A-C Patchy and Flat **D, E** landscapes with *E. coli* and *P. aeruginosa* mixed 1:1 prior to inoculation at one focal point **A, D**; two focal points **B**; and distributed along the whole landscape **C, E**.

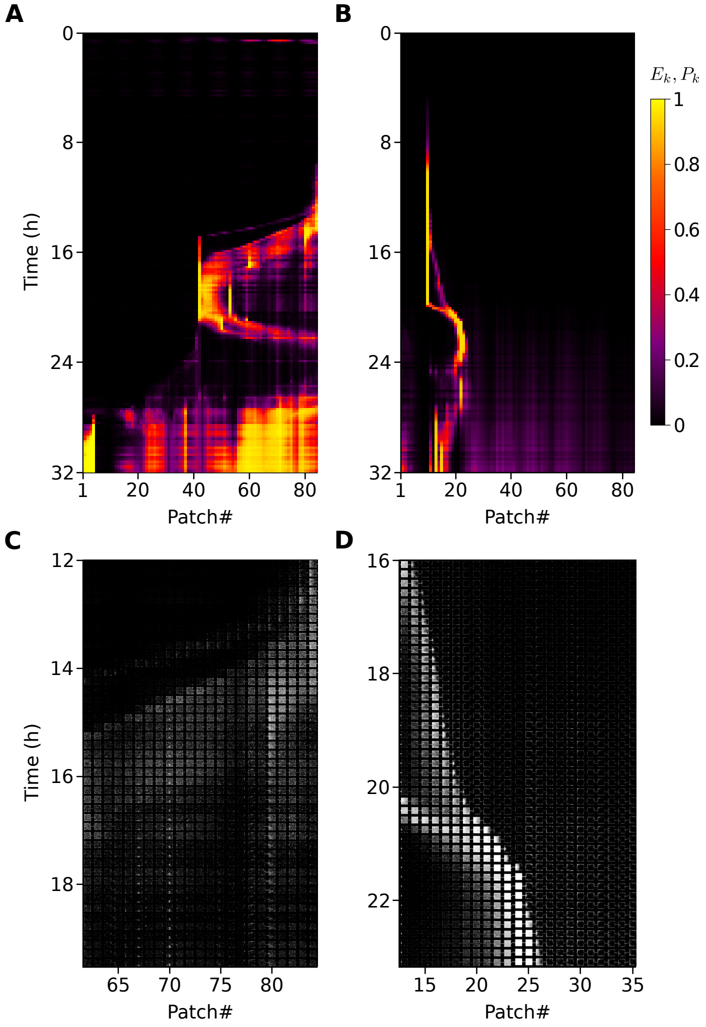

Figure S4: Monoculture experiments in patchy landscape A, C Colonization of a patchy landscape *on-chip* and zoom in view of population waves by *E . coli* and **B, D** *P. aeruginosa* metapopulations.

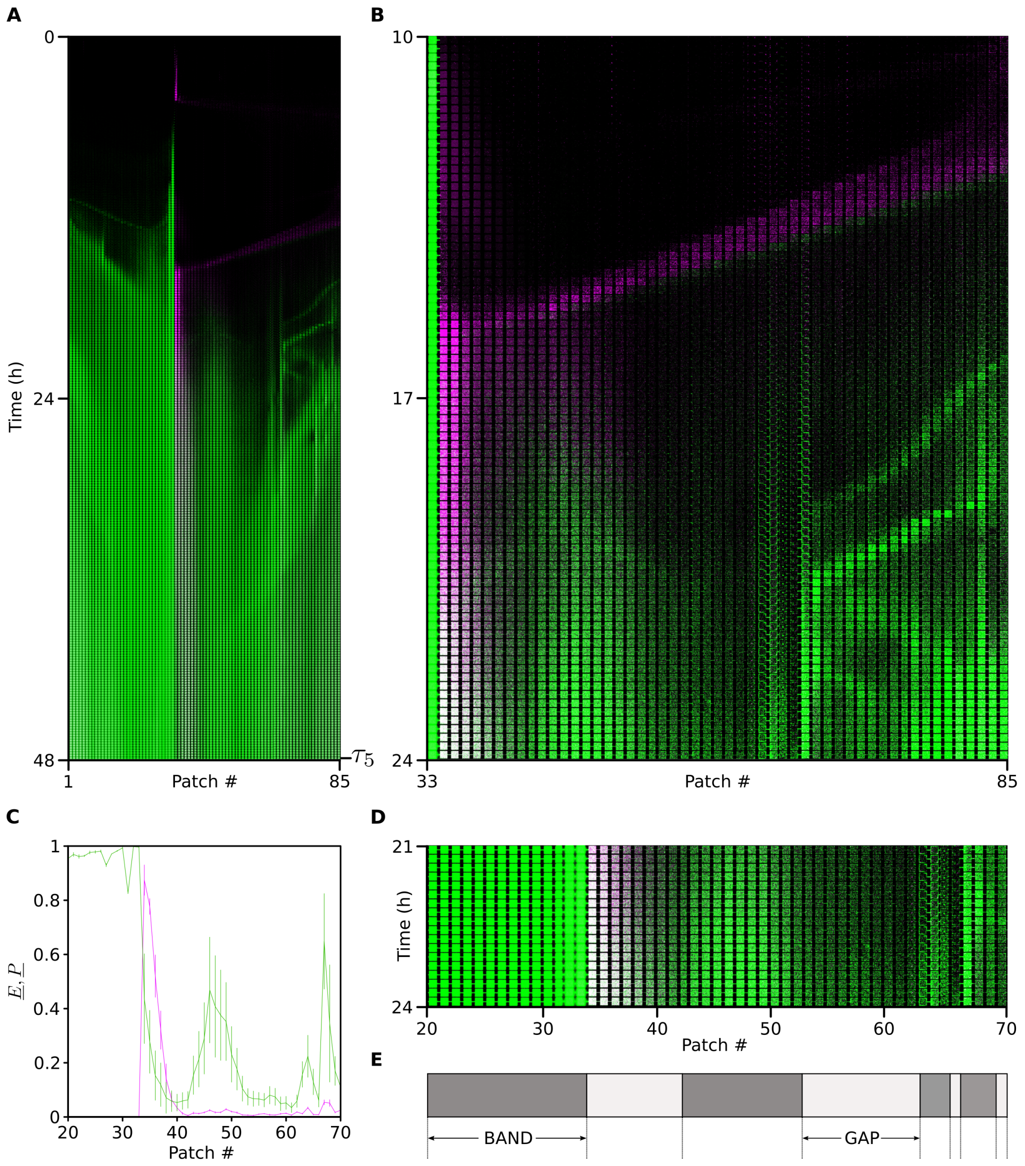

Figure S5: Structure and dynamics of a bacterial metacommunity **A** Complete (85 MHPs; 48 hrs) kymograph partially shown in Figure 4 C,D. **B** A Zoom-in to a region of space ($33 \leq k \leq 85$) and time ($10 \leq t \leq 24$) of (A) highlighting wave dynamics and ecological succession. **C** Average of 3 hours of occupancy data for *E. coli* ($\underline{\text{E}}_k = (18)^{-1} \sum_{21<t\leq 24} E_k(t)$; magenta) and *P. aeruginosa* ($\underline{\text{P}}_k = (18)^{-1} \sum_{21<t\leq 24} P_k(t)$; green) for each MHP ($20 \leq k \leq 70$) shown in **D** ROI of kymograph shown in (A). **E** Band gap structure of *P. aeruginosa*'s P_k in (D).

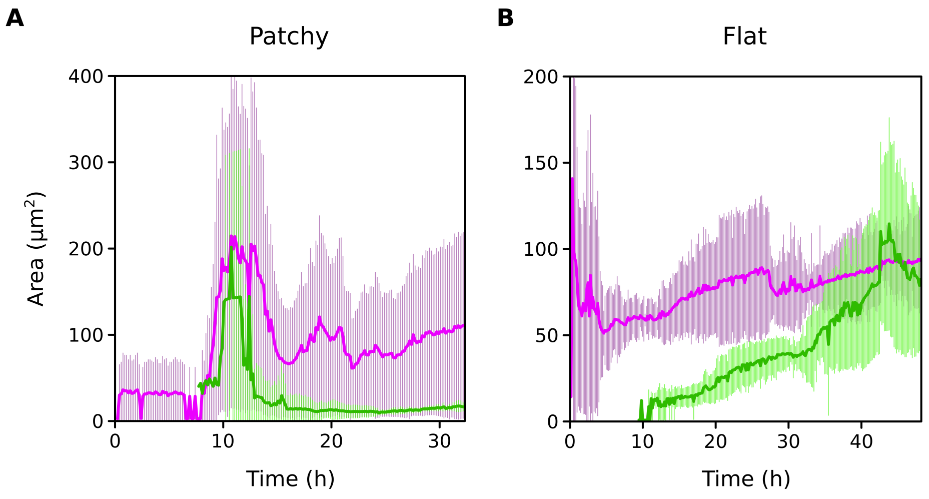

Figure S6: Micro-colony structure in patchy versus flat landscapes A B Dynamics of the size of micro-colonies for *E.coli* (*P.aeruginosa*) in magenta (green) for monoculture experiments in patchy landscapes (A) and flat landscapes (B).

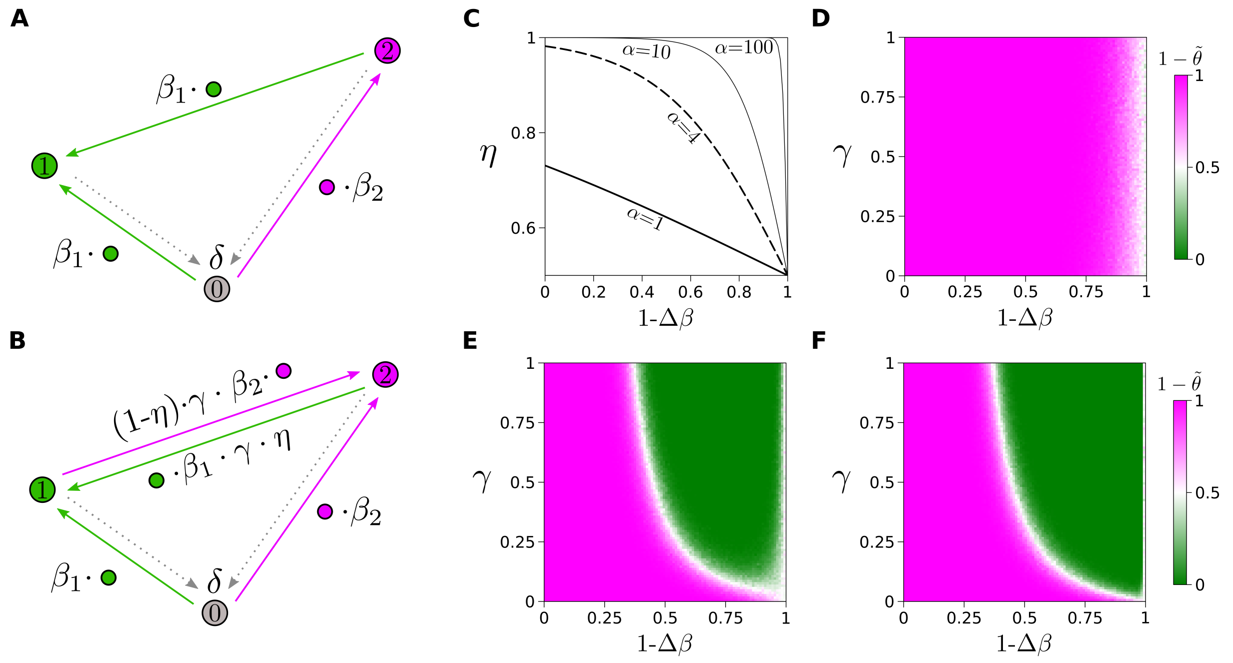

Figure S7: Models of the competition-colonization (CC) trade-off A Markov chain of the CC model (*1*). **B** Markov chain of the Calcagno model (*2*). **C** Strength of interference competition advantage, η, expressed as a trade-off with competitive ability, $(1 - \Delta\beta)$, for four different strengths, α, of the trade-off. **D-F** Our spatial (2D; $r = 1$) model's parameter space $[\gamma, (1 - \Delta\beta)]$ for three of the trade-off strength values shown in D which are $\alpha = 1$ (D), $\alpha = 10$ (E), and $\alpha = 100$ (F); value of $\alpha = 4$ is shown in Figure 5B.

Table S1: Symbols

Symbol	Meaning
**Experiments**	
$k \in \{1 \cdots 85\}$	Index of a Micro fabricated Habitat Patch (MHP);
E_k, P_k	Local occupancy within MHP number k
	The symbols E_k and P_k correspond to
	E. coli and *P. aeruginosa* respectively.
$k_s \in \{1 \cdots 85\}; s \in \{1 \cdots 72\}$	index for MHP number k in landscape s
E_{k_s}, P_{k_s}	Local occupancy within MHP k_s in array s
$\bar{E}_s, \bar{P}_s$	Spatial average across MHPs (k) in landscape s;
	$\bar{E}_s = (1/85) \cdot \sum_{k_s} E_{k_s}$ and $\bar{P}_s = (1/85) \cdot \sum_{k_s} P_{k_s}$.
$Z_{k_s} = (E_{k_s} + P_{k_s})/2$	Normalized total occupancy in MHP k_s in array s
$\Theta_{k_s} = P_{k_s}/(E_{k_s} + P_{k_s})$	Fractional occupancy of *P. aeruginosa* in MHP k_s in array s
$(1 - \Theta_{k_s}) = E_{k_s}/(E_{k_s} + P_{k_s})$	Fractional occupancy of *E. coli* in MHP k_s in array s
$\Theta_s = \bar{\Theta}_{k_s}$	*P. aeruginosa* fractional occupancy averaged across MHPs in s
$(1 - \Theta_s) = 1 - \bar{\Theta}_{k_s}$	*E. coli* fractional occupancy averaged across MHPs in s
$\Theta = \langle \Theta_s \rangle$	Ensemble average of Θ_s across all arrays (n=72)
N_E, N_P	Number of cells of *E. coli* (N_E) and *P. aeruginosa* (N_P)
θ	Fractional density of *P. aeruginosa* in well-mixed environments
	averaged across experiments $\theta \equiv \langle N_P/(N_E + N_P) \rangle$ (n=3)
t	Time t in experiments is a full-landscape image scan
τ_j, τ'_j	A time point $t = \tau_j$ or a binned collection of successive times τ'_j
**Theory**	
$\Delta\omega$	Difference in interference competition ability
$\Delta\beta$	Difference in scramble competition ability; $\Delta\beta = \beta_2 - \beta_1$
α	Strength of the trade-off model between competitive abilities
$\xi_t : \mathcal{L} \to \mathcal{S}$	Spatial stochastic process describing the state of sites at time t
$\mathcal{L}$	Lattice sites $x, y \in \mathcal{L}$ can be arranged in 1D ($\mathcal{L}_{1\text{D}}$) or 2D ($\mathcal{L}_{2\text{D}}$)
	with boundary conditions, $\mathcal{L}_{1\text{D}} \cong Z_{100}$ and $\mathcal{L}_{2\text{D}} \cong Z^2_{100\times100}$
$\mathcal{S} = \{0, 1, 2, *\}$	Set of states. Each lattice site can be in a state of this set
$\Omega(x; r)$	Interaction neighborhood $\Omega \in \mathcal{L}$ with radius r around x
	so we have that $\Omega(x; r) = \{y \in \mathcal{L} :\mid x - y \mid \leq r\} \subset \mathcal{L}$
β_1, β_2	Colonization rates indexed such $\beta_1 < \beta_2 \equiv 1$
	Competitor type (state 1) represents *P. aeruginosa*
	and colonizer type (state 2) corresponds to *E. coli*.
$(1 - \Delta\beta) = \beta_1$	Superior (interference) competitor's colonization ability
γ	Degree of priority effects (rate of co-colonization modulation)
η	Interference competition bias on a localized competitive lottery
δ	Clearance rate (local extinction) of a sub-population
ρ_P, ρ_E	Proportions of sites held by type 1 (ρ_P) or type 2 (ρ_E)
$\tilde{\theta} = \rho_P/(\rho_E + \rho_P)$	Fractional occupancy of competitor type (state 1)
$(1 - \tilde{\theta}) = \rho_E/(\rho_E + \rho_P)$	Fractional occupancy of the colonizer type (state 2)
$(1 - \tilde{\theta}_t) = \rho_E(t)/[\rho_E(t) + \rho_P(t)]$	Dynamics of colonizer's fractional occupancy

Table S2: Glossary

Term	Meaning	Refs.
**Biophysics**		
bacterial waves	Motile bacteria swim together as cell packs	(*3, 4*)
α-wave	First, fast, low density wave of bacteria	(*3, 5*)
microfluidics	Manipulation of small volumes of fluids	
microfabrication	Using (soft/hard) lithography techniques to build microfluidics devices	(*6*)
arrays of MHPs	Using microfabrication and microfluidics to capture patchiness in microbial habitats	(*7*)
localized cells	Non traveling "resident" cells (sessile or planktonic)	
**Microbiology**		
bacterial chemotaxis	Motile bacteria responds to chemical fields	(*3*)
biofilm	Development program; bacteria attached to surfaces	(*8*)
lag phase (LAG)	First phase of colony development with no growth	
log phase (LOG)	Logarithmic growth phase of a culture/colony	
stationary phase (SP)	Phase where growth comes to a halt	
sessile	Cells aggregated to surfaces/each other	
planktonic	Cells swimming in the liquid interface	
**Ecology**		
landscape ecology	Scaling ecology from locality to landscapes	(*9*)
habitat patch	Spatially discrete, ephemeral, favorable conditions	
patchy landscapes	Habitats which are collections of patches.	
ecotope	Ecologically unique unit of a landscape	(*10*)
occupancy (vacancy)	Local patches, are occupied or vacant	
lattice model (IPS)	Spatially explicit models of site occupancy	(*11*)
metapopulation	A population of populations	(*12*)
metacommunity	A collection of metapopulations of $\neq$ species	(*13*)
CC trade-off	Trade-off between competition and colonization	(*14*)
fugitive strategies	A life history focused on scramble competition	(*15*)

Table S3: Bacterial strains

Strain ID	Plasmid	Characteristics	Reference
E. coli			
JEK1036		W3110; lacZY::GFPmut2	(*16*)
JEK1037		W3110; lacZY::mRFP1	(*16*)
P. aeruginosa			
PUPa3-G	pKR-C12	PUPa3; Gm^R Amp^R;<br>pBBR1MCS-5 carrying PlasB–gfp(ASV) Plac–lasR	(*17*), (*18*)
PUPa3-R	pKR-C12	PUPa3; Gm^R Amp^R;<br>pBBR1MCS-5 carrying PlasB–dsRed(ASV)Plac–lasR	(*17*)

References and Notes

1. Kinzig A, Levin SA, Dushoff J, Pacala S. Limiting similarity, species packing, and system stability for hierarchical competition-colonization models. Am Nat. 1999;153(4):371–383.

2. Calcagno V, Mouquet N, Jarne P, David P. Coexistence in a metacommunity: the competition–colonization trade-off is not dead. Ecol Lett. 2006;9(8):897–907.

3. Adler J. Chemotaxis in Bacteria. Science. 1966;153(3737):708–716. Available from: https://science.sciencemag.org/content/153/3737/708.

4. Saragosti J, Calvez V, Bournaveas N, Perthame B, Buguin A, Silberzan P. Directional persistence of chemotactic bacteria in a traveling concentration wave. Proc Natl Acad Sci U S A. 2011;108(39):16235–16240.

5. Van Vliet S, Hol FJ, Weenink T, Galajda P, Keymer JE. The effects of chemical interactions and culture history on the colonization of structured habitats by competing bacterial populations. BMC Microbiol. 2014;14(1):116.

6. Qin D, Xia Y, Whitesides GM. Soft lithography for micro-and nanoscale patterning. Nat Protoc. 2010;5(3):491.

7. Keymer JE, Galajda P, Muldoon C, Park S, Austin RH. Bacterial metapopulations in nanofabricated landscapes. Proc Natl Acad Sci U S A. 2006;103(46):17290–17295.

8. O'Toole G, Kaplan HB, Kolter R. Biofilm formation as microbial development. Annu Rev Microbiol. 2000;54(1):49–79.

9. Wiens JA. Spatial Scaling in Ecology. Funct Ecol. 1989;3(4):385–397. Available from: http://www.jstor.org/stable/2389612.

10. Whittaker RH, Levin SA, Root RB. Niche, habitat, and ecotope. Am Nat. 1973;107(955):321–338.

11. Durrett R. Stochastic spatial models. SIAM Rev Soc Ind Appl Math. 1999;41(4):677–718.

12. Levins R. Some demographic and genetic consequences of environmental heterogeneity for biological control. American Entomologist. 1969;15(3):237–240.

13. Leibold MA, Holyoak M, Mouquet N, Amarasekare P, Chase JM, Hoopes MF, et al. The metacommunity concept: a framework for multi-scale community ecology. Ecol Lett. 2004;7(7):601–613.

14. Levins R, Culver D. Regional coexistence of species and competition between rare species. Proc Natl Acad Sci U S A. 1971;68(6):1246–1248.

15. Horn HS, MacArthur RH. Competition among fugitive species in a harlequin environment. Ecology. 1972;53(4):749–752.

16. Keymer JE, Galajda P, Lambert G, Liao D, Austin RH. Computation of mutual fitness by competing bacteria. Proc Natl Acad Sci U S A. 2008;105(51):20269–20273.

17. Kumar RS, Ayyadurai N, Pandiaraja P, Reddy A, Venkateswarlu Y, Prakash O, et al. Characterization of antifungal metabolite produced by a new strain Pseudomonas aeruginosa PUPa3 that exhibits broad-spectrum antifungal activity and biofertilizing traits. J Appl Microbiol. 2005;98(1):145–154.

18. Riedel K, Hentzer M, Geisenberger O, Huber B, Steidle A, Wu H, et al. N-acylhomoserine-lactone-mediated communication between Pseudomonas aeruginosa and Burkholderia cepacia in mixed biofilms. Microbiology. 2001;147(12):3249–3262.
