# Supplementary material for: Ecological succession and the competition-colonization trade-off in microbial communities
Source: BMC Biol. 2022 Nov 30;20:262. doi: 10.1186/s12915-022-01462-5 (PMC9710175; doi:10.1186/s12915-022-01462-5)
Supplement: Supplementary file 2 — Additional file 2: Supplementary Note 1. Alternative initial conditions inoculating with a 1:1 mixture of species. Supplementary Note 2. Stochastic spatial model. Supplementary Note 3. Patch occupancy and the competition-colonization trade-off. [file 12915_2022_1462_MOESM2_ESM.pdf]

Ecological succession and the competition-colonization trade-off in microbial communities

Miles T. Wetherington [1,2,3], Krisztina Nagy [2], László Dér [2],
Ágnes Ábrahám [2,4], Janneke Noorlag [1,6], Peter Galajda [2]
and Juan E. Keymer [1,5,6]

[1]Department of Ecology, School of Biological Sciences, P. Catholic University of Chile
[2]Biological Research Centre, Institute of Biophysics
[3] School of Applied and Engineering Physics, Cornell University
[4]Doctoral School of Multidisciplinary Medical Sciences, University of Szeged
[5]Institute of Physics, School of Physics, P. Catholic University of Chile
[6]Department of Natural Sciences and Technology, University of Aysén

Additional file 2

Supplementary Note 1: Alternative initial conditions inoculating with a 1:1 mixture of species

An interesting alternative initial condition to evaluate in microfluidics experiments is to inoculate devices with a 1:1 mix of *E. coli* and *P. aeruginosa* at some location. In Additional file 1: Fig. S3 we show experiments with three patterns of inoculating a 1:1 mixture of the bacterial species as initial conditions for our spatial competition experiments: (*i*) flat landscape and patchy landscape - single inlet (*ii*) patchy landscape - both inlets (*iii*) flat landscape and patchy landscape - distributed across the entire landscape. In Additional file 1: Fig. S3C *E. coli* is completely outcompeted by *P. aeruginosa* in the long term under initial condition (*iii*). On the contrary, when inoculating the patchy landscape from either one (Additional file 1: Fig. S3A) or both (Additional file 1: Fig. S3B) inlets, *E. coli* is able to escape competition by migrating away from the local community

where it finds itself in a 1:1 mixture with *P. aeruginosa*. Although in a more precarious situation than in our previous (main text) experiments, as it is in local competition at initial condition, and even though there is no priority effect at the location of inoculation, very quickly *E. coli* is able to colonize empty space away from *P. aeruginosa*'s competition. The same is true for the flat landscapes. When a single inlet is inoculated (Additional file 1: Fig. S3D), *E. coli* can out-migrate *P. aeruginosa* and reach a location not yet reached by the superior competitor. If inoculation is along the whole landscape (Additional file 1: Fig. S3E), *P. aeruginosa* dominates, and *E. coli* can be seen only when it enters the landscape as transient incursions from the inlets. In short, the more spatial resources (vacant sites, not inoculated locations) are available at the beginning, regardless of local competition at the inoculation site, *E. coli* is capable of escaping competition by colonization. If the landscape holds less vacancy (due to a spatially extended initial inoculation), the less colonization potential there is for the fugitive *E. coli*. In summary, these results complement our experiments inoculating landscapes at each of the inlets with pure cultures of each species and show that our main results are not an artifact of the inoculation pattern, but a consequence of the available vacancy in the habitat (at inoculation time) which can be exploited by the fugitive colonizer.

Supplementary Note 2: Stochastic spatial model

We consider a spatial stochastic process $\xi_t : \mathcal{L} \to \mathcal{S} = \{0, 1, 2, *\}$ (*1*). Thus, a lattice site $x \in \mathcal{L}$ at time t is in state $\xi_t(x) = s_x \in \mathcal{S}$. These states are vacancy {0}, occupancy {1, 2}, and localized co-occupancy {*}. Our model is ruled by reactions which take place only if lattice sites are adjacent. Such adjacency dependence in our model is controlled by a radius r for which the interaction neighborhood $\Omega(x, r)$ is defined. Thus, interactions take place only if the site y representing the source of propagules is in the vecinity $\Omega(x, r)$ of the focal site x being colonized, $\mid x - y \mid \leq r; \forall x, y \in \mathcal{L}$.

Reactions (Fig. 5A) correspond to (*i*) a disturbance/clearance rate where all occupied sites turn vacant at rate $\delta \equiv 0.1$; (*ii*) a difference in the colonization ability $\Delta\beta$ between fast $\beta_2 \equiv 1$ and slow $\beta_1 = 1 - \Delta\beta$ (dispersal) strategies; (*iii*) a competition bias $\eta = (1 + e^{\alpha \cdot \Delta\beta})^{-1}$ in favor of the superior competitor; and (*iv*) a priority effect parameter γ controlling the existence of the mix state.

Particle extinction $s_x \to 0_x$, affecting occupied sites ($s_x \neq 0$), occurs at a rate δ which does not depend on the state of near-by sites. An event (local extinction) occurring at rate δ, means that the times Δt_i between successive occurrences (lifespan of the occupied states) has an exponential distribution with parameter δ; that is, $\mathcal{P}(\Delta t_i \leq t) = 1 - \exp(-\delta t)$. Colonization reactions all depend on the state of near-by sites as they are the sources of colonization propagules. We assume that only states 1 and 2 can spread the process in space by wave emission. The mixed state $*$ is assumed to be localized and with no emissions so it cannot infect near-by sites. This co-occupancy state represents co-localized populations of competitors engaged only in interference competition. Vacant sites (0_x) can be colonized, $0_x + s_y \to s_x + s_y$, at rate β_{s_y}/z by propagules coming from near-by sites in state $s_y \in \{1, 2\}$ where z is the number of neighbors to x within $\Omega(x, r)$. Sites occupied by type i can be cross-colonized by type $j \neq i$ by reaction $i_x + j_y \to *_x + j_y$ or $j_x + i_y \to *_x + i_y$ occurring at rate $\gamma \cdot \beta_{s_y}/z$ for $s_y \in \{1, 2\}$ (priority effects). When a site is co-occupied, the local community is localized and its members cannot spread to near-by sites. State $*$ decays to either of the non-localized types (state 1 or 2) by a reaction representing the competitive lottery $*_x \to s_x \in \{1, 2\}$ occuring at rate η for type 1 and at rate $(1 - \eta)$ for type 2 and which is determined by Equation 3. We also considered a spatial version of Equation 4 by ignoring the mixed state $*$ and consider a restricted set of states $\mathcal{S} = \{0, 1, 2\}$ defined by implementing the transitions shown in Additional file 1: Fig. S7C as lattice reactions of an interacting particle system (*2*).

Supplementary Note 3: Patch occupancy and the competition-colonization trade-off

Our model suggests a simple but universal trade-off between competition and colonization for space to address the successional nature of such ecosystems and the limits of similarity in niche space between coexisting species. A large body of theoretical work on this model often focuses on the long-term outcome of community dynamics considering one exogenously driven local extinction rate (δ) but species specific colonization rates (β) (*3–6*).

In the strict hierarchical form of the CC model, two species are ranked $i = 1, 2$ according to their competitive abilities, which are opposite to their colonization rates, $\beta_1 < \beta_2$, such that locally the superior competitor (type 1) always out-competes the inferior (type 2) at each site. Tracking site occupancy p_i,

$$\frac{\dot{p}_1}{p_1} = \beta_1(1 - p_1) - \delta \qquad (1)$$

$$\frac{\dot{p}_2}{p_2} = \beta_2(1 - p_1 - p_2) - \delta - \beta_1 p_1, \qquad (2)$$

we represent such a system in a Mean Field (MF) approximation. In this strict MF scenario, interference competition within sites happens instantaneously, such that the superior competitor 'senses' a completely vacant habitat represented by Equation 1. The inferior competitor on the other hand, must persist in a dynamic landscape imposed by the superior competitor (*7*).

Extensions to this theory address some of the more unrealistic aspects of the CC model while still maintaining its generality, notably *i*) the deterministic nature of the strict competitive hierarchy and *ii*) the equivalency of colonizing vacant versus occupied patches. Calcagno and collaborators (*8*) addressed these issues. First, by relaxing the strict competitive hierarchy, which by itself has been shown to make coexistence unlikely (*9*). Furthermore, they introduced a form of priority effect ($\gamma \in [0, 1]$), such that the prior arrival of a species to a patch would affect future colonization events by competitors. A competitive index ω can be defined to depend on colonization differ-

ences $\Delta\beta$ between the life history strategies of our two bacterial species (Eq. 1, main text). Such CC trade-off relationship (*10*) is plotted in Figure 1D and can be linked to community dynamics as follows: After patches enter a short lived state of co-occupancy, they can be won back by a competitive lottery parameter

$$\eta_{i,j} = \frac{\omega_i}{\omega_i + \omega_j} = \frac{e^{-\alpha\cdot\beta_i}}{e^{-\alpha\cdot\beta_i} + e^{-\alpha\cdot\beta_j}} \tag{3}$$

which determines mean field meta-community dynamics as a balance between scramble and interference competition,

$$\frac{\dot{p}_i}{p_i} = \underbrace{\beta_i \left(1 - \sum_{i\neq 0} p_i\right) - \delta}_{\text{scramble}} + \underbrace{\gamma \sum_{j\neq i\neq 0} \left(\beta_i \eta_{i,j} - \beta_j \eta_{j,i}\right) p_j}_{\text{interference}} . \tag{4}$$

Unlike in the CC model with strict competitive hierarchy (Eqs. 1 and 2) where competition is instantaneous (Additional file 1: Fig. S7A); in the model of Calcagno and collaborators (Eq. 4), resident species are not immediately overwhelmed by a superior competitor (Additional file 1: Fig. S7B). Instead, they can invade an occupied site with some probability γ while the resident is still present, after which a biased competitive lottery representing interference competition ruled by competitive parameter $\eta_{i,j}$ (Eq. 3) determines which type succeeds in claiming the site. For two species, we simply have $\eta_{1,2} = 1 - \eta_{2,1} \equiv \eta$. From Equation 3 we can relate such competition bias η to differences in colonization ability $\Delta\beta$ between our bacterial species using the CC trade-off relationship (Eq. 1, main text) for a given strength α (Additional file 1: Fig. S7C-F). If there are no differences ($\Delta\beta = 0$), then the competitive ability $\eta = 0.5$ regardless of the value of α. A value $\alpha = 0$ implies that η is the same regardless of differences in colonization, while a value $\alpha = \infty$ on the other hand implies an absolute competitive hierarchy *sensu* (*5*). These qualities of a system represented by Equation 4 reflect our experimental observations, wherein a MHP first colonized by *E. coli* and eventually invaded by *P. aeruginosa*, often gets taken over by the latter.

However, spatially explicit interactions are missing in this model. When comparing a spatial

construction based on Equation 4 lacking a mixed state with our model including one, an important difference surfaces: the mixed state allows for the separation of scramble and interference interactions. A consequence of this separation is that the mixed state sequesters occupied sites from participating in scramble competition. Akin to habitat destruction/fragmentation, mixed state site sequestration acts as a mechanism for the 'extinction debt' of the superior competitor (*11*) beyond a critical level of priority effects $\gamma \to \gamma_c$.

References and Notes

1. Durrett R. Stochastic spatial models. SIAM Rev Soc Ind Appl Math. 1999;41(4):677–718.
2. Liggett TM. Interacting particle systems. vol. 276. Springer Science & Business Media; 2012.
3. Levins R, Culver D. Regional coexistence of species and competition between rare species. Proc Natl Acad Sci U S A. 1971;68(6):1246–1248.
4. Horn HS, MacArthur RH. Competition among fugitive species in a harlequin environment. Ecology. 1972;53(4):749–752.
5. Hastings A. Disturbance, coexistence, history, and competition for space. Theoretical Population Biology. 1980;18(3):363–373.
6. Tilman D. Competition and biodiversity in spatially structured habitats. Ecology. 1994;75(1):2–16.
7. Kinzig A, Levin SA, Dushoff J, Pacala S. Limiting similarity, species packing, and system stability for hierarchical competition-colonization models. Am Nat. 1999;153(4):371–383.
8. Calcagno V, Mouquet N, Jarne P, David P. Coexistence in a metacommunity: the competition–colonization trade-off is not dead. Ecol Lett. 2006;9(8):897–907.
9. Yu DW, Wilson HB. The competition-colonization trade-off is dead; long live the competition-colonization trade-off. Am Nat. 2001;158(1):49–63.
10. Geritz SA, van der Meijden E, Metz JA. Evolutionary dynamics of seed size and seedling competitive ability. Theor Popul Biol. 1999;55(3):324–343.
11. Tilman D, May RM, Lehman CL, Nowak MA. Habitat destruction and the extinction debt. Nature. 1994;371(6492):65–66.
